# Digital Gene Expression Analysis Provides Insight into the Transcript Profile of the Genes Involved in Aporphine Alkaloid Biosynthesis in Lotus (*Nelumbo nucifera*)

**DOI:** 10.3389/fpls.2017.00080

**Published:** 2017-01-31

**Authors:** Mei Yang, Lingping Zhu, Ling Li, Juanjuan Li, Liming Xu, Ji Feng, Yanling Liu

**Affiliations:** ^1^Key Laboratory of Aquatic Plant and Watershed Ecology, Wuhan Botanical Garden, Chinese Academy of SciencesWuhan, China; ^2^Department of Agricultural Sciences, Viikki Plant Science Center, University of HelsinkiHelsinki, Finland; ^3^College of Life Science, University of Chinese Academy of SciencesBeijing, China; ^4^Tobacco Research Institute of Hubei ProvinceWuhan, China

**Keywords:** lotus, aporphine alkaloids, co-expression network, biosynthesis, putative genes

## Abstract

The predominant alkaloids in lotus leaves are aporphine alkaloids. These are the most important active components and have many pharmacological properties, but little is known about their biosynthesis. We used digital gene expression (DGE) technology to identify differentially-expressed genes (DEGs) between two lotus cultivars with different alkaloid contents at four leaf development stages. We also predicted potential genes involved in aporphine alkaloid biosynthesis by weighted gene co-expression network analysis (WGCNA). Approximately 335 billion nucleotides were generated; and 94% of which were aligned against the reference genome. Of 22 thousand expressed genes, 19,000 were differentially expressed between the two cultivars at the four stages. Gene Ontology (GO) enrichment analysis revealed that catalytic activity and oxidoreductase activity were enriched significantly in most pairwise comparisons. In Kyoto Encyclopedia of Genes and Genomes (KEGG) analysis, dozens of DEGs were assigned to the categories of biosynthesis of secondary metabolites, isoquinoline alkaloid biosynthesis, and flavonoid biosynthesis. The genes encoding norcoclaurine synthase (NCS), norcoclaurine 6-O-methyltransferase (6OMT), coclaurine N-methyltransferase (CNMT), N-methylcoclaurine 3′-hydroxylase (NMCH), and 3′-hydroxy-N-methylcoclaurine 4′-O-methyltransferase (4′OMT) in the common pathways of benzylisoquinoline alkaloid biosynthesis and the ones encoding corytuberine synthase (CTS) in aporphine alkaloid biosynthetic pathway, which have been characterized in other plants, were identified in lotus. These genes had positive effects on alkaloid content, albeit with phenotypic lag. The WGCNA of DEGs revealed that one network module was associated with the dynamic change of alkaloid content. Eleven genes encoding proteins with methyltransferase, oxidoreductase and CYP450 activities were identified. These were surmised to be genes involved in aporphine alkaloid biosynthesis. This transcriptomic database provides new directions for future studies on clarifying the aporphine alkaloid pathway.

## Introduction

Lotus belongs to the genus *Nelumbo*, the sole member of the Nelumbonaceae family. This genus consists of two species, *Nelumbo nucifera* (Asia, Australia, Russia) and *Nelumbo lutea* (North America) (Xue et al., 2012). It is an ornamental plant and an important economical crop in Asian countries, especially in China. Lotus is characterized by dainty flowers, round leaves, ellipsoidal seeds and fleshy rhizomes, thus it has been cultivated as ornamental or vegetable plant for 7000 years throughout Asia, for its beautiful flowers and its edible rhizomes and seeds (Shen-Miller, 2002; Zhang et al., 2011). In addition, lotus has religious significance in both Buddhism and Hinduism throughout the history because of its pure and sacred meaning. Virtually, as a source of herbal medicine, every part of the lotus plant, including its leaves, buds, flowers, anther, stamens, fruit, stalks, and roots, have been used for treatment of various diseases, such as pectoralgia, liver disease, heart disease, cancer, insomnia, diabetes, obesity, and hypertension (Lacour et al., 1995; Kashiwada et al., 2005; Ono et al., 2006; Huang et al., 2010; Nguyen et al., 2012; Zhang et al., 2012; Poornima et al., 2014). Alkaloids are the most important active components in lotus, more than 20 had been identified to date (Zhu et al., 2011; Nakamura et al., 2013). On the basis of their structures, alkaloids in lotus can be divided into three categories: aporphines, bisbenzylisoquinolines, and monobenzylisoquinolines. Aporphine alkaloids accumulate mainly in leaves, and include nuciferine, O-nornuciferine, N-nornuciferine, anonaine, and roemerine (Luo et al., 2005; Itoh et al., 2011; Chen et al., 2013b; Nakamura et al., 2013). This accumulation starts from the early developmental stages, peaks when the leaves reaches its full size, and then decreases slightly during senescence (Deng et al., 2016). The bisbenzylisoquinoline alkaloids, including liensinine, isoliensinine, and neferineare, accumulate predominantly in the seed embryo (Itoh et al., 2011; Chen et al., 2013b; Deng et al., 2016). Monobenzylisoquinoline alkaloids are intermediate products in the biosynthesis of aporphine and bisbenzylisoquinoline alkaloids, and occur in trace amounts in several lotus organs (Itoh et al., 2011; Nakamura et al., 2013).

All of the aporphine, bisbenzylisoquinoline, and monobenzylisoquinoline alkaloids belong to the benzylisoquinoline alkaloids (BIAs). BIAs are a large and diverse group of natural products found primarily in several plant families, and include approximately 2500 defined structures (Ziegler and Facchini, 2008; Chow and Sato, 2013; Glenn et al., 2013). BIAs are not essential for normal growth and development but appear to function in the defense of plants against herbivores and pathogens. Many of the estimated BIAs are pharmacologically active. The most prominent compounds are the narcotic analgesic morphine, the vasodilator papaverine, the potential anti-cancer drug noscapine, and the antimicrobial agents sanguinarine and berberine (Liscombe and Facchini, 2008; Ziegler and Facchini, 2008; Chow and Sato, 2013; Glenn et al., 2013). Alkaloids in lotus have pharmacological effects such as anti-hypertensive, anti-obesity, anti-cancer, and anti-human immunodeficiency virus (HIV) activities (Lacour et al., 1995; Kashiwada et al., 2005; Ono et al., 2006; Huang et al., 2010; Nguyen et al., 2012; Poornima et al., 2014). Owing to these pharmacological properties, lotus has received increasing attention in recent years.

**Figure 1:**
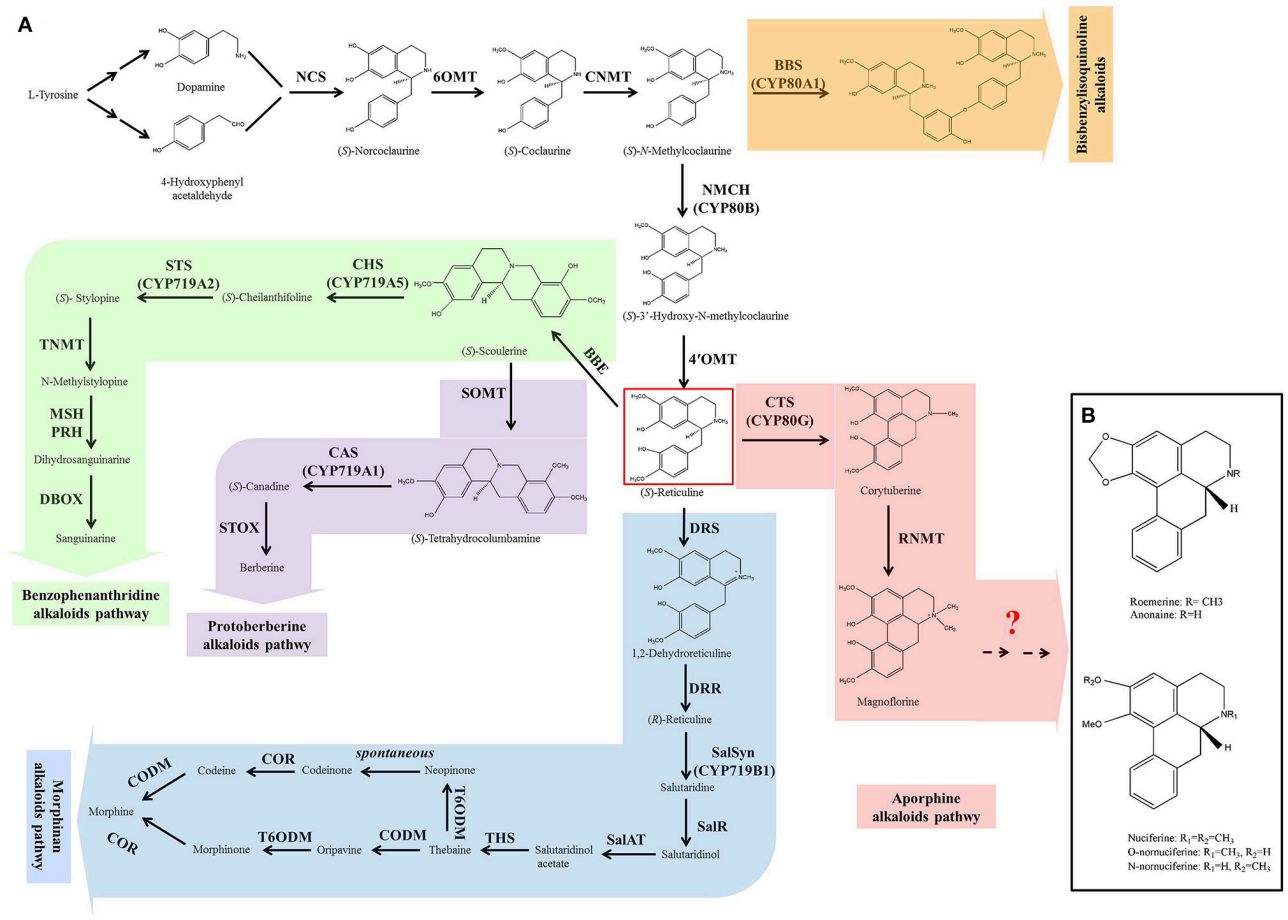
**The biosynthesis of benzylisoquinoline alkaloids (BIAs) in plant (A)** and the structures of five prominent aporphine alkaloids in lotus **(B)**. The biosynthesis of BIAs includes five branch pathways: aporphine, benzophenanthridine, morphinan, protoberberine, and bisbenzylisoquinoline alkaloids. These branch pathways are synthesized through a common pathway derived from L-tyrosine. The pathways are color-coded: white, common pathway; pink, aporphine alkaloids; green, benzophenanthridine alkaloids; blue, morphinan alkaloids; purple, protoberberine alkaloids; and orange, bisbenzylisoquinoline alkaloids. Abbreviations of the enzymes for these pathways: BBE, berberine bridge enzyme; BBS, berbamunine synthase; CAS, canadine synthase; CHS, cheilanthifoline synthase; CNMT, coclaurine N-methyltransferase; CODM, codeine 3-O-demethylase; COR, codeinone reductase; CTS, corytuberine synthase; DBOX, dihydrobenzophenanthridine oxidase; DRR, 1,2-dehydroreticuline reductase; DRS, 1,2-dehydroreticuline synthase; MSH, N-methylstylopine 14-hydroxylase; NCS, norcoclaurine synthase; NMCH, N-methylcoclaurine 3′-hydroxylase; 4′OMT, 3′-hydroxy-N-methylcoclaurine 4′-O-methyltransferase; 6OMT, norcoclaurine 6-O-methyltransferase; PRH, protopine 6-hydroxylase; SalAT, salutaridinol-7-O-acetyltransferase; SalR, salutaridine:NADPH 7-oxidoreductase; SalSyn, salutaridine synthase; SOMT, scoulerine-9-O-methyltransferase; STOX, (*S*)-tetrahydroprotoberberine oxidase; STS, stylopine synthase; THS, thebaine synthase; TNMT, tetrahydroprotoberberine cis-N-methyltransferase; T6ODM, thebaine 6-O-demethylase.

Although BIAs show wide structural diversity, their biosynthetic pathways are all initiated by the condensation of dopamine and 4-hydroxyphenyl acetaldehyde, which is catalyzed by norcoclaurine synthase (NCS) (Figure 1A). Then, a series of enzymes, norcoclaurine 6-O-methyltransferase (6OMT), coclaurine N-methyltransferase (CNMT), (*S*)-N-methylcoclaurine-3-hydroxylase (NMCH), and 3-hydroxy-N-methylcoclaurine 4′-O-methyltransferase (4′OMT), exhibit catalytic activity to yield the central branch point intermediate (*S*)-reticuline. (*S*)-Reticuline undergoes diverse reactions resulting in the formation of a wide variety of backbone structures, including morphinan (morphine), benzophenanthridine (sanguinarine), protoberberine (berberine), and aporphine alkaloid subclasses (Liscombe and Facchini, 2008; Ziegler and Facchini, 2008; Chow and Sato, 2013; Glenn et al., 2013; Hagel and Facchini, 2013). Bisbenzylisoquinoline alkaloids are not produced via (*S*)-reticuline, but are intermediates from the (*S*)-reticuline precursor N-methylcoclaurine, which is catalyzed by the P450 enzyme CYP80A1 (Kraus and Kutchan, 1995; Ziegler and Facchini, 2008). The common pathway (from the initial step to the produce of (*S*)-reticuline) and the five branches of morphinan, benzophenanthridine, protoberberine, and bisbenzylisoquinoline have been characterized clearly in Opium poppy (*Papaver somniferum*), *Eschscholzia californica*, Japanese goldthread (*Coptis japonica*) and yellow meadow rue (*Thalictrum flavum*). Most of the genes encoding the enzymes in these pathways have been isolated (Liscombe and Facchini, 2008; Ziegler and Facchini, 2008; Chow and Sato, 2013; Glenn et al., 2013; Hagel and Facchini, 2013). However, little is known about aporphine alkaloid biosynthesis.

The first committed step in the aporphine synthetic pathway begins with the conversion of (*S*)-reticuline to corytuberine, which is catalyzed by corytuberine synthase (CTS) (Liscombe and Facchini, 2008; Chow and Sato, 2013). CTS has been isolated and characterized in *C. japonica* (Ikezawa et al., 2008). Then, reticuline N-methyltransferase (RNMT) catalyzes corytuberine to yield magnoflorine by C8-C2 coupling, and has been cloned in *P. somniferum* (Morris and Facchini, 2016). However, in lotus, it remains unclear how the five aporphine alkaloids: nuciferine, O-nornuciferine, N-nornuciferine, anonaine, and roemerine are yielded from corytuberine and magnoflorine. Structural analysis of these five aporphine alkaloids suggests that they are produced from corytuberine and/or magnoflorine through oxidation, dehydroxylation, and demethylation (Figure 1B). These processes require oxidoreductases, methyltransferases, and demethylase. Dissecting these late steps is a key to understand the biosynthetic pathway of aporphine alkaloids in lotus.

Besides the enzymatic genes in the BIA biosynthetic pathway, two transcription factors regulate the BIA metabolism. A WRKY (*CjWRKY1*) is the first discovered transcription factor involved in the coordinate regulation of BIA biosynthesis. Its expression caused a substantial increase in the expression of several berberine biosynthetic genes in opium poppy (Kato et al., 2007). The ectopic expression of *Arabidopsis WRKY1* increase morphinan alkaloid content in California poppy cells (Apuya et al., 2008). Transient RNA interference and overexpression of *CjbHLH1*, a novel basic helix–loop–helix protein in *C. japonica*, revealed the regulation of *CjbHLH* in transcription of berberine biosynthetic genes. *CjbHLH1* had similar activity to *CjWRKY1* (Yamada et al., 2011).

RNA-seq is a valuable tool for transcriptomic profiling, such as digital gene expression (DGE) technology (Glaser, 2009; Wang et al., 2009; Asmann et al., 2012). Recently, DGE has been used widely to detect differentially expressed genes (DEGs) in crops, such as rice (Ding et al., 2016), maize (Eveland et al., 2010; Liu et al., 2015), cotton (Wei et al., 2013) and *Brassica* (Jiang et al., 2015); in woody plants, such as peach (Li et al., 2015), orange (Yang et al., 2013; Sun et al., 2014), pear (Zhang et al., 2015), white spruce (Albouyeh et al., 2010), and *Metasequoia glyptostroboides* (Zhao et al., 2015); and in orchids, such as cymbidium (Yang and Zhu, 2015), *Rosa chinensis* (Yan et al., 2015), and *Tagetes erecta* (Ai et al., 2016). DGE promotes our understanding of the major changes in metabolic processes and enables us to detect DEGs. For example, DGE analysis have been used successfully to predict biosynthetic pathways of the alkaloids, rhynchophylline and isorhynchophylline, in *Uncaria rhynchophylla*, a non-model plant with potent anti-Alzheimer's properties (Guo et al., 2014), which suggests that DGE analysis can be used to understand alkaloid biosynthesis in lotus.

We sequenced the genome of an ancient lotus cultivar, “China Antique” (Ming et al., 2013), and performed transcriptome profiling analysis to investigate the expression of genes related to floral transition and rhizome development in lotus (Yang et al., 2014, 2015). These provide a solid foundation for the use of DGE analysis to understand the transcript profiling of the genes involved in alkaloid biosynthesis in lotus. Building on this previous work, here, we performed DGE analysis of two lotus cultivars, “10–48” (a low-leaf -alkaloid cultivar) and “Luming Lian” (a high-leaf-alkaloid cultivar). We analyzed four leaf development stages: leafbud (S1), curly leaf underwater (S2, the day before leaf emergence from water), unfolded leaf (S3, day 4 after leaf emergence from water, the beginning of leaf expansion), and mature leaf (S4, day 20 after leaf emergence from water), with three biological repeats. By examining the levels of gene expression digitally, we identified the DEGs among the cultivars and stages. Using weighted gene co-expression network analysis (WGCNA), the co-expression networks were constructed and the potential genes responsible for the late steps in aporphine alkaloid biosynthesis were predicted. These results lay a foundation for further characterization of the aporphine alkaloid pathway in lotus.

## Materials and methods

### Plant materials

Two cultivars of *N. nucifera*, “10–48” and “Luming Lian,” were used for DGE analysis. “10–48” is a low-alkaloid cultivar, and “Luming Lian” is a high-alkaloid cultivar. They were planted in a trial plot at Wuhan Botanical Garden, Chinese Academy of Sciences, Hubei Province, China on 13 April, 2012. Each cultivar was planted in a separate cement pool of 25 m^2^ (5 × 5 m) and supplemented with 20-cm-deep paddy soil and 1 kg organic fertilizer (mature soybean seedcake) before planting. A total of 200 g chemical fertilizer was top dressed at both the early developmental stage of standing leaves and the initial stage of flowering. Throughout the growth season, the depth of water in the pool was maintained at 20–25 cm, with an average temperature of 30°C during the day and 20°C at night.

**Figure 2:**
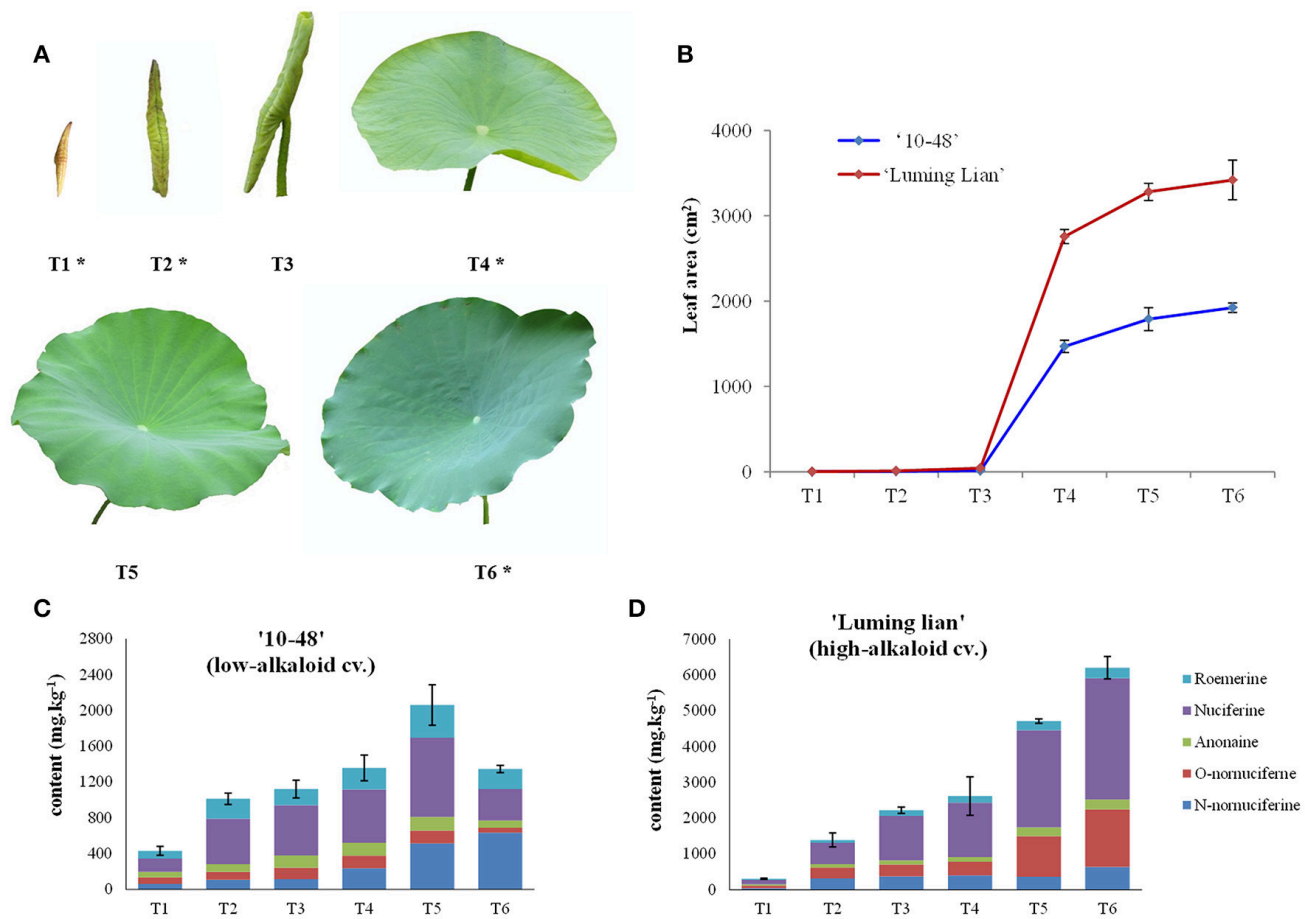
**Leaf development and alkaloid content of “10–48” (low-alkaloid cv.) and “Luming Lian” (high-alkaloid cv.). (A)** Six leaf developmental stages, leafbud (T1), curly leaf underwater (T2, the day before leaf emergence from water), curly leaf emergence from water (T3, day 2 after leaf emergence from water), unfolded leaf (T4, day 4 after leaf emergence from water, the beginning of leaf expansion), semi-mature leaf (T5, day 10 after leaf emergence from water, leaf area does not increase) and mature leaf (T6, day 20 after leaf emergence from water). The leaves at the stages T1, T2, T4, and T6 are selected for DGE analysis and are indicated with an asterisk, being renamed as S1, S2, S3, and S4, respectively. **(B)** Leaf area at different developmental stages for “10–48” and “Luming Lian.” **(C)** Five profiles of alkaloids, roemerine, anonaine, nuciferine, O-nornuciferine, and N-nornuciferine at different leaf developmental stages for “10–48.” **(D)** Five profiles of alkaloids, roemerine, anonaine, nuciferine, O-nornuciferine, and N-nornuciferine at different developmental stages of leaf for “Luming Lian.”

Leaves from “10–48” and “Luming Lian” were collected at six stages: leafbud (T1), curly leaf underwater (T2, the day before leaf emergence from water), curly leaf emergence from water (T3, day 2 after leaf emergence from water), unfolded leaf (T4, day 4 after leaf emergence from water, the beginning of leaf expansion), semi-mature leaf (T5, day 10 after leaf emergence from water, leaf area does not increase) and mature leaf (T6, day 20 after leaf emergence from water), throughout the growth season (Figure 2A). Analysis of leaf development and alkaloid content for the two cultivars identified four stages, T1, T2, T4, and T6, that would be particularly appropriate for transcriptome sampling, which were renamed S1, S2, S3, and S4, respectively. The leaves from each stage were collected from three comparable plants used as three biological replicates, transferred immediately to liquid nitrogen, and then stored at −80°C until RNA extraction.

Alkaloid content of the leaves at each developmental stage was determined using the HPLC-MS, as described by Chen et al. (2013b). Alkaloid was extracted from approximately 600 mg of fresh leaves with 30 mL methanol–1.0% hydrochloric acid aqueous solution (1:1, v/v) by ultrasonication for 30 min, followed by centrifugation at 15,000 × g for 5 min. The supernatant was then collected and diluted to 50 mL in a volumetric flask. 2 mL of each solution was passed through a 0.22-μm filter and 10 μL of the filtered solution was used for HPLC analysis. A-eluent was Milli-Q water containing 1%0; (v/v) triethylamine, and B-eluent was acetonitrile. The gradient profile started with 40–80% B at 0–15 min, followed by 80% B at 18 min, 80–40% B at 19 min, and then equilibration of the column at 40% B for 3 min; all at a flow rate of 0.8 mL/min with a column temperature at 30°C. The chromatograms were acquired at 272 nm. The UV-vis and photodiode array spectra were recorded from 180 to 400 nm.

### RNA extraction and sequencing

Total RNA was extracted using the Easyspin RNA reagent (RN38; Aidlab Biotechnologies, Beijing, China), and treated with RNase-free DNase I (Takara, Dalian, China) to remove genomic DNA contamination. The RNA integrity was evaluated with a 1.0% agarose gel stained with ethidium bromide. The quality and quantity of RNA were assessed using a NanoPhotometer® spectrophotometer (Implen, Westlake Village, CA, USA) and an Agilent 2100 Bioanalyzer (Agilent Technologies, Santa Clara, CA, USA). The RNA integrity number was >8.0 for all samples.

The RNA samples were later used to construct cDNA libraries and Illumina sequences which were completed by Beijing Novogene Bioinformatics Technology Co. Ltd (Beijing, China). Poly(A) mRNA was prepared and sequences from each of the four developmental stages were indexed with unique nucleic acid identifiers. Library quality was assessed on the Agilent Bioanalyzer 2100 system. The libraries were sequenced on an Illumina Hiseq 2000 platform and 100-bp paired-end reads were generated. All raw-sequence reads data were deposited in NCBI Sequence Read Archive (SRA, http://www.ncbi.nlm.nih.gov/Traces/sra) with accession number SRP095042.

### Analysis of RNA-Seq data

After removing those reads with only adaptor and unknown nucleotides >5%, or those that were of low quality (*Q* < 20), the clean reads were filtered from the raw reads, then aligned to the reference genome sequence (Ming et al., 2013) using the program Tophat version 2.0.9 (Trapnell et al., 2012). The tolerance parameters were the default settings, allowing mismatches of no more than two bases. For inclusion in the calculation of Reads Per Kilobases per Millionreads (RPKM) values (Mortazavi et al., 2008), cut-offs were set such that ≥50% of a read in contiguous nucleotides must be aligned to the reference transcript with ≥98% identity. When reads could be mapped to multiple reference locations, they were assigned to reference transcripts that were based proportionally on the relative number of unique reads.

The DESeq package was used to detect DEGs between the two samples (Anders and Huber, 2010). The false discovery rate (FDR) was used to determine the *P*-value threshold in multiple tests. FDR ≤ 0.005 and absolute value of the log_2_(fold change) with RPKM ≥ 1 were used as the thresholds to determine significant differences in gene expression (Benjamini and Yekutieli, 2001). The heatmap of DEGs was produced with Cluster 3.0 (de Hoon et al., 2004). Before cluster formation, RPKM expression values for each transcript were normalized to between −2.0 and 2.0 by log-transformation. The heatmap was clustered using the complete linkage hierarchical analysis based on Euclidean distance.

Functional enrichment analyses, including Gene Ontology (GO) and Kyoto Encyclopedia of Genes and Genomes (KEGG) were performed to identify which GO terms or metabolic pathways were significantly associated with the DEGs. The GO enrichment analysis of DEGs was implemented using the GOseq R package, in which gene length bias was corrected. GO terms with a corrected *P* ≤ 0.05 were considered significantly enriched among the DEGs. The GO annotations were classified functionally using WEGO software for gene function distributions. KOBAS software was used to test the statistical enrichment of DEGs in KEGG pathways. The pathways with an FDR ≤ 0.05 were defined as those with genes that show significant levels of differential expression.

### Phylogenetic analysis of the genes

Protein sequences of the genes that are known to be involved in BIA biosynthesis were used for phylogenetic analysis. Sequence alignment was performed using ClustalW in MEGA5 (Tamura et al., 2011) and adjusted manually, as necessary. The resulting data were analyzed using the neighbor-joining method. The bootstrap values were calculated from 1000 replicates.

### Gene co-expression network analysis

The highly co-expressed gene modules were inferred from the DEGs using WGCNA, an R package (Langfelder and Horvath, 2008). WGCNA network construction and module detection were conducted using an unsigned type of topological overlap matrix (TOM), a power β of 10, a minimal module size of 30, and a branch merge cut-off height of 0.25. The most significant correlated genes with WGCNA edge weight >0.15 were visualized using Cytoscape 3.3 (Kohl et al., 2011).

### Quantitative real-time PCR validation of RNA-Seq data

Seven genes involved in the pathways of BIAs biosynthesis and 11 candidate genes were selected for validation using quantitative real-time PCR (RT-qPCR). Primers for RT-qPCR, which were designed with the Primer 3.0 software (http://biotools.umassmed.edu/bioapps/primer3_www.cgi), are listed in Supplementary Table 1. RT-qPCRs were analyzed in the ABI StepOne™ Plus Real-Time PCR System with the SYBR Green PCR Master Mix (Takara), and amplified with 1 μL cDNA template, 5 μL 2 × SYBR Green Master Mix, and 0.2 μL each primer (10 μmol/μL), to a final volume of 10 μL by adding water. The amplification program consisted of one cycle of 95°C for 10 s, followed by 40 cycles of 95°C for 15 s and 60°C for 60 s. Fluorescent products were detected in the last step of each cycle. Melting curve analysis was performed at the end of 40 cycles to ensure proper amplification of target fragments. All RT-qPCR procedures for each gene were performed in three biological replicates, with three technical repeats per experiment. Relative gene expression was normalized by comparison with the expression of lotus β*-actin* (NNU24864), and analyzed using the 2^−ΔΔCT^ method (Livak and Schmittgen, 2001). The data are presented as means ± SE (*n* = 9). Statistical analysis of RT-qPCR data was conducted using the ANOVA procedure of SAS 8.1 (SAS Institute, Cary, NC, USA).

## Results

### Alkaloid content in leaves at different developmental stages

Leaves from “10–48” and “Luming Lian” were sampled at six stages (T1–T6, Figure 2A). Five profiles of alkaloids: roemerine, anonaine, nuciferine, O-nornuciferine, and N-nornuciferine, were tested for the two cultivars at the six stages (Figures 2C,D). Nuciferine and N-nornuciferine were the dominant compounds for “10–48,” and nuciferine and O-nornuciferine were the dominant compounds for “Luming Lian.” Total alkaloid content of “10–48” increased from 430.0 to 2060.7 mg kg^−1^ in stages T1–T5, and decreased to 1346.1 mg kg^−1^ at T6. Total alkaloid content of “Luming Lian” increased rapidly throughout the period, ranging from 308.6 to 6203.0 mg kg^−1^. In the first two stages, there was no significant difference in total alkaloid content between the two cultivars, and from stages T3 to T6, total alkaloid content of “Luming Lian” was higher than that of “10–48.” Therefore, total alkaloid content of “Luming Lian” at T6 was 4.6 times higher than that of “10–48” (Figures 2C,D). Given our interest in the changes in expression of genes involved in alkaloid metabolic pathways, based on these data, we chose the stages T1, T2, T4, and T6 for DEG analysis. Leaves from these four stages of “10–48” and “Luming Lian” were sampled to construct eight cDNA libraries: LS1–4 and HS1–4.

### HiSeq mRNA sequencing and number of expressed genes

Three biological repeats were set for each stage of the two cultivars, and 24 samples were sequenced (Supplementary Table 2). Every sample generated ≥6 million nucleotides of sequence data. Therefore, 335 billion nucleotides were obtained from these samples. Ninety-four percent of the short clean reads were aligned against the lotus “China Antique” reference genome (Ming et al., 2013). To verify the reproducibility of the sequencing data, Person's correlation coefficients for three biological replicates at each stage were calculated by log_10_RPKM. All biological replicates had a strong correlation (*R*^2^ ≥ 0.97) except for the library HS1R3 (Supplementary Table 3).

Among the 26,685 annotated genes, 22,893 and 22,782 genes were expressed in the low-alkaloid cultivar “10–48” and high-alkaloid cultivar “Luming Lian,” respectively. Of these expressed genes, 22,225 were detected in both cultivars. Only 668 and 557 genes were specifically expressed in “10–48” and “Luming Lian,” respectively (Supplementary Figure 1A). Approximately 22,000 genes were expressed in each of the samples, and 21,000 genes were co-expressed in three biological repeats for each stage of the two cultivars (Supplementary Table 2). Of these co-expressed genes, there were approximately 18,000 genes that were commonly expressed in the four stages for each cultivar (Supplementary Figures 1B,C). The following analyses were based on the co-expressed genes in three biological repeats for each stage of the two cultivars.

### DEGs during leaf development

**Figure 3:**
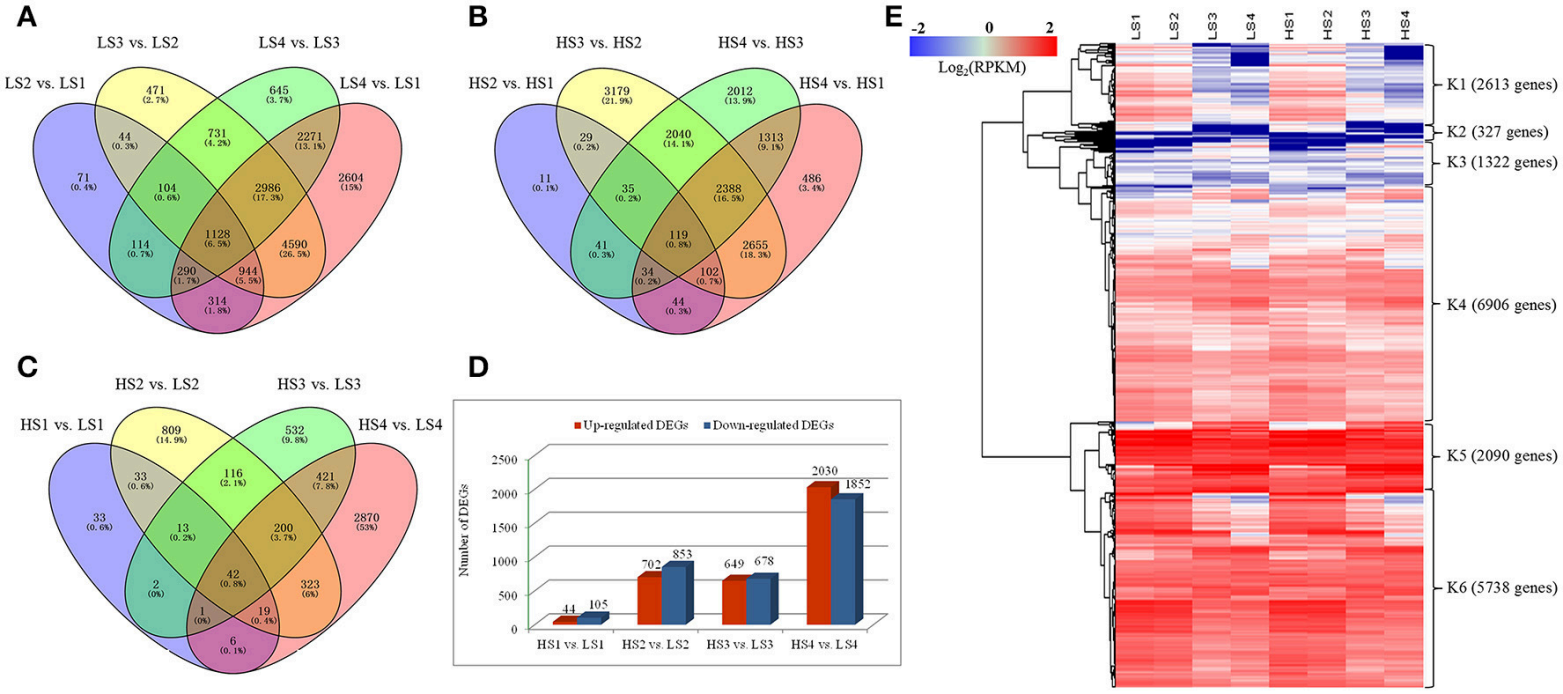
**Venn diagram and cluster analysis of differentially expressed genes (DEGs) identified by pairwise comparisons of the four stages in the two cultivars. (A)** Venn diagram of DEGs in “10–48” (low-alkaloid cv.) by pairwise comparisons of the four stages. **(B)** Venn diagram of DEGs in “Luming Lian” (high-alkaloid cv.) by pairwise comparisons of the four stages. **(C)** Number of DEGs between “10–48” and “Luming Lian” at the particular stage. The separate and overlapping areas in Venn diagrams represent the numbers of specifically expressed and co-expressed genes between different stages, respectively. **(D)** Numbers of up-regulated and down-regulated DEGs between “10–48” and “Luming Lian” at the particular stage. **(E)** Heatmap of DEGs across the four developmental stages in the lotus cultivars, “10–48” and “Luming Lian.” Expression values are presented as RPKM normalized log_2_transformed counts. Red and blue colors indicate up- and down- regulated transcripts, respectively.

Differences in gene expression at the four leaf development stages in the two cultivars were examined, and DEGs were identified by pairwise comparisons of these libraries (Figure 3). The total number of DEGs across the four stages was higher in “10–48” than in “Luming Lian” at each stage. Comparisons of the four stages of “10–48” revealed 3009, 10,998, 8269, and 15,127 DEGs in the pairs LS2 vs. LS1, LS3 vs. LS2, LS4 vs. LS3, and LS4 vs. LS1, respectively. Comparisons of the four stages of “Luming Lian” revealed 415, 5434, 7982, and 7141 DEGs in the pairs of HS2 vs. HS1, HS3 vs. HS2, HS4 vs. HS3, and HS4 vs. HS1, respectively (Figures 3A,B). The number of DEGs detected in same-stage comparisons between the two lotus cultivars was generally lower than that detected from same-cultivar comparisons at different stages. Comparisons of the same stage between the two lotus cultivars identified 149, 1555, 1327, and 3882 DEGs in the pairs HS1 vs. LS1, HS2 vs. LS2, HS3 vs. LS3, and HS4 vs. LS4, respectively (Figure 3C). The number of the up-regulated DEGs was larger than that of the down-regulated ones in the pairs HS1 vs. LS1 and HS2 vs. LS2, but lower than that of the down-regulated DEGs in HS4 vs. LS4 (Figure 3D).

There were 19,000 genes which were expressed differentially for “10–48” and “Luming Lian” at the four stages. We performed hierarchical clustering of these DEGs using the Euclidean distance method associated with complete-linkage. The expression patterns of six clusters, K1–K6, were plotted (Figure 3E). The K1 cluster included 2613 genes that showed down-regulation in both cultivars as the stages progressed, and the expression levels of these genes were low at the S3 and S4 stages. Most of the genes in the K2 cluster, despite being up-regulated as development progressed, had low expression levels. Most of the genes in K3 and K4 were up-regulated as the stages progressed in both cultivars, and the levels of expression of the genes in K4 were higher than those in K3. The clusters K5 and K6 possessed 7828 DEGs, that were significantly down-regulated as the stages progressed in both cultivars, and the expression of genes in K5 was higher than those in K6. Overall, the levels of expression of genes in K5 and K6 were higher than those in K1–K4 (Figure 3E).

### Functional classification of DEGs during leaf development

**Figure 4:**
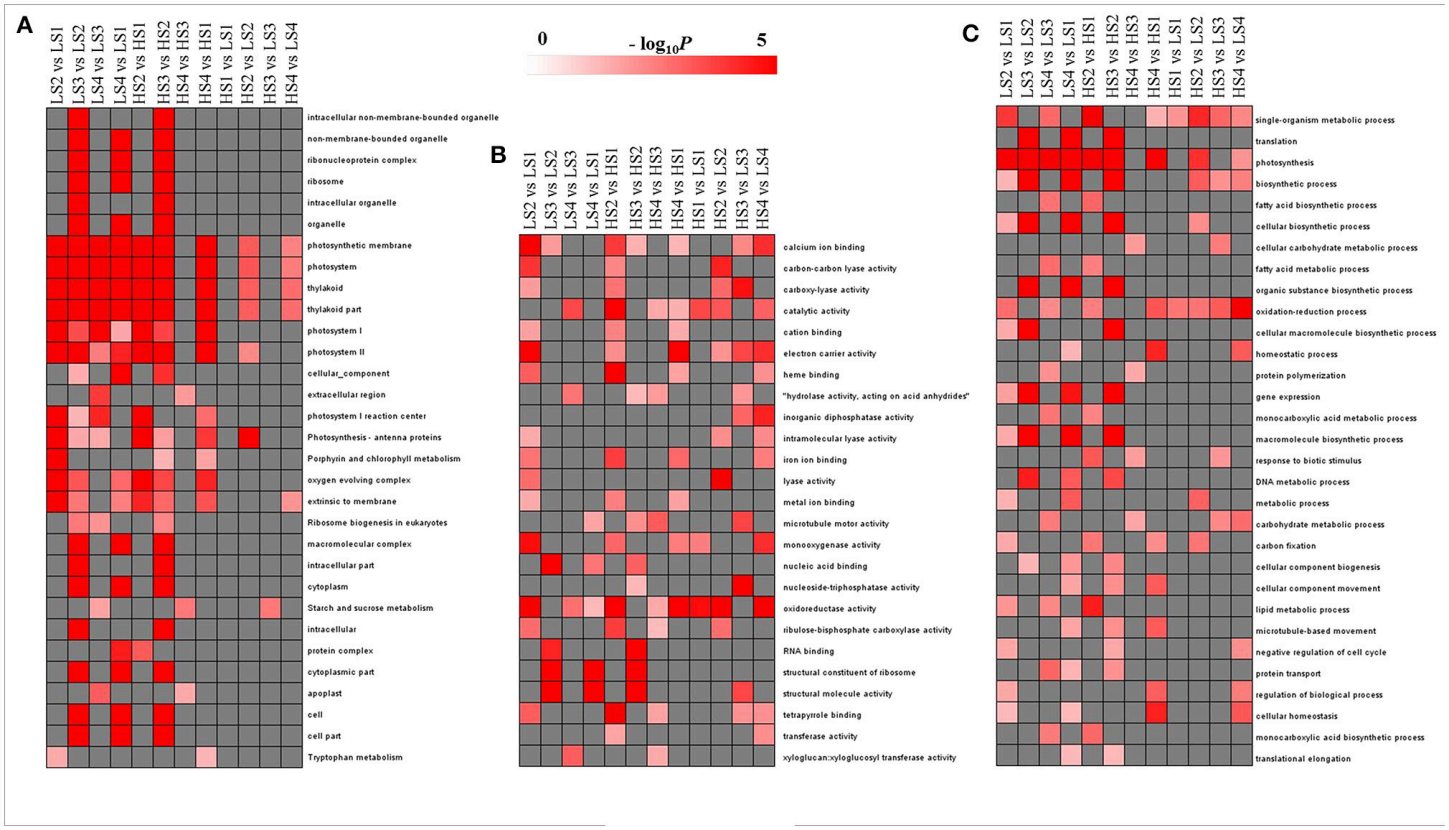
**GO-term function enrichment analysis of pairwise comparisons: (A)** cellular component, **(B)** molecular function, and **(C)** biological process. The significance of the most represented GO Slims in each comparison pair is indicated using log-transformed *P*-value (red); the dark gray areas represent missing values.

To evaluate the potential functions of DEGs, GO assignments were used to classify the functions of DEGs in pairwise comparisons of the library between two cultivars and between four developmental stages. The overrepresented GO terms of DEGs in the three GO categories (cellular component, molecular function, and biological process) are listed in Figure 4. In the cellular component category, no GO terms were enriched significantly in HS1 vs. LS1 and HS3 vs. LS3. The other pairwise comparisons had high proportions of transcripts associated with photosystem and thylakoid. The enriched GO terms in the comparisons of two cultivars at each stage were fewer than those across the stages for each cultivar (Figure 4A). In the molecular function category, no GO terms were enriched significantly in all pairwise comparisons, but catalytic activity and oxidoreductase activity were enriched significantly in most pairwise comparisons (Figure 4B). In the biological process category, three GO terms, single-organism metabolic process, photosynthesis, and oxidation–reduction process, were enriched significantly in most comparisons. There were fewer enriched GO terms in same-stage comparisons between the two lotus cultivars than those across the stages for each cultivar (Figure 4C).

**Figure 5:**
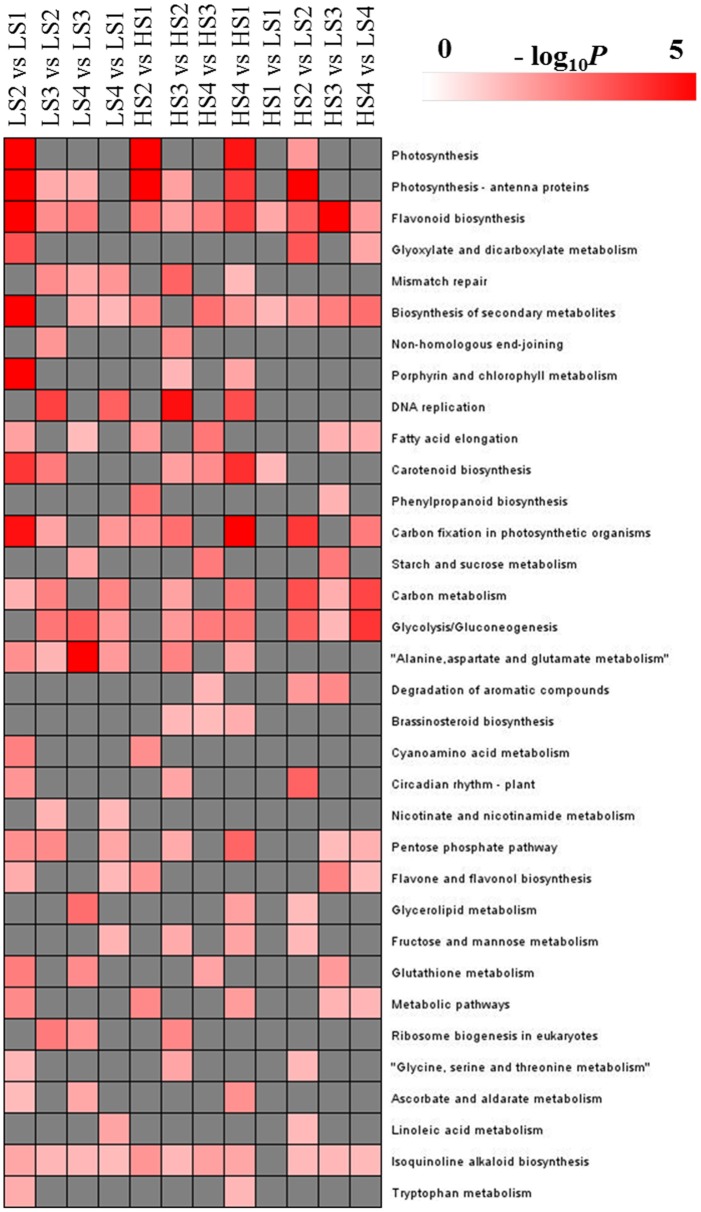
**KEGG pathways that were significantly enriched in pairwise comparisons**. The significance of the most strongly represented pathway in each comparison pair is indicated using log-transformed *P*-value (red); the dark gray areas represent missing values.

KEGG analysis was performed to explore the pathways in which DEGs were involved (Figure 5). Dozens of DEGs in all pairwise comparisons were assigned to the biosynthesis of secondary metabolites, isoquinoline alkaloid biosynthesis, and flavonoid biosynthesis. BIA biosynthesis is part of isoquinoline alkaloid biosynthesis, and belongs to the category of biosynthesis of secondary metabolites. Therefore, the transcriptomic profiling analysis could provide new information to characterize aporphine alkaloid biosynthesis.

### Genes in the BIAs biosynthetic pathway

The biosynthesis of BIAs includes the common pathway and five branch pathways: aporphine, benzophenanthridine, morphinan, protoberberine, and bisbenzylisoquinoline alkaloids, which are synthesized through a common pathway derived from L-tyrosine (Figure 1). We identified only 23 genes involved in the common pathway, aporphine, morphinan and protoberberine pathways. There are four homologs for NCS, four for 6OMT, one for CNMT, two for NMCH, four for 4'OMT, and two for CTS, three for scoulerine-9-O-methyltransferase (SOMT), one for codeine 3-O-demethylase (CODM), and two for thebaine 6-O-demethylase (T6ODM).

**Figure 6:**
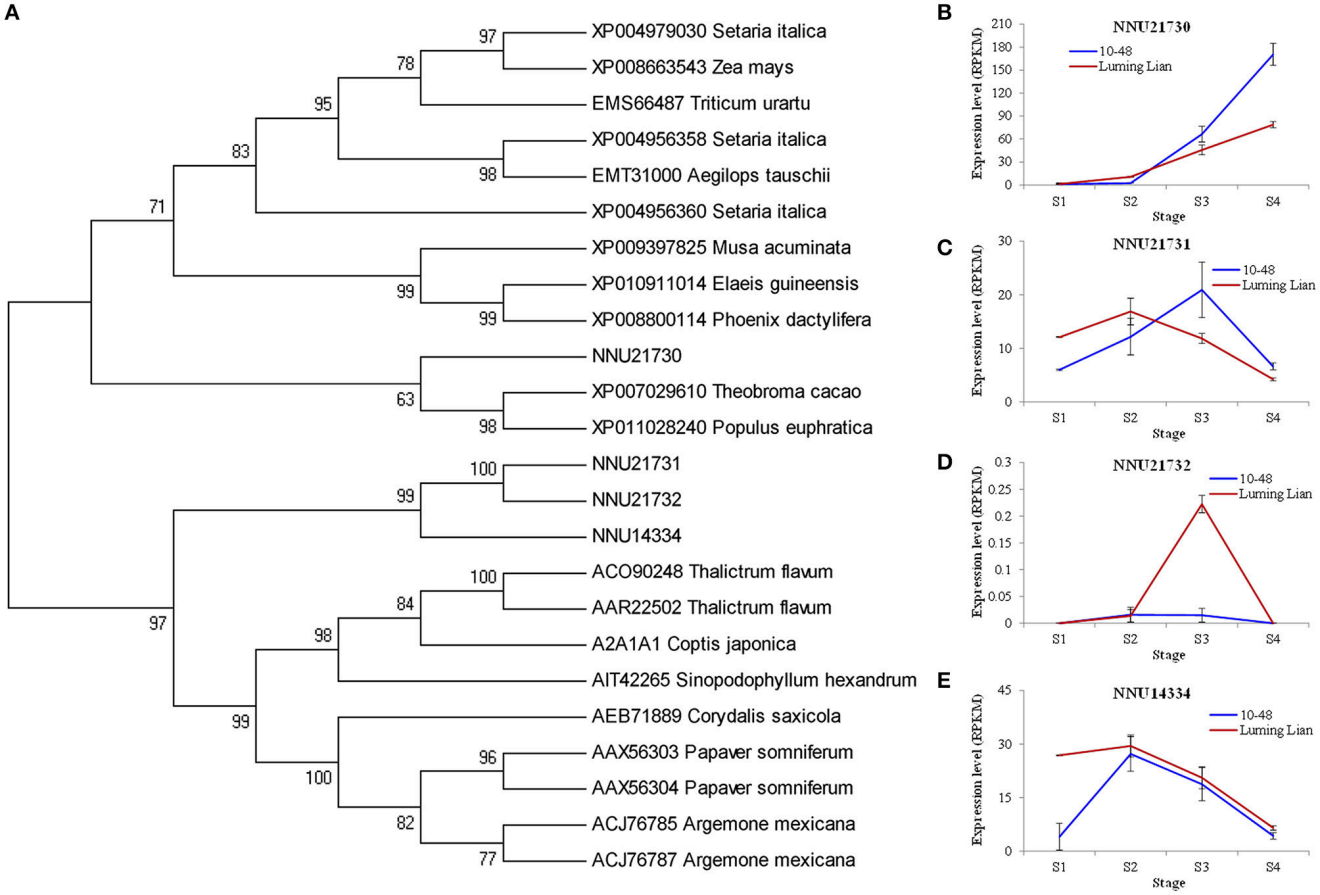
**Phylogenetic relationship of norcoclaurine synthase (NCS) for different species based on protein sequence (A)** and the expression of four lotus *NCS* genes at four developmental stages in two lotus cultivars **(B–E)**. In **(A)**, the value at each node is the bootstrap support value from 1000 replicates. In **(B–E)**, RPKM value for each gene used three biological replicates; the error bars indicate standard error (*n* = 3).

NCS is the first committed enzyme in BIA biosynthesis, which catalyzes the condensation of dopamine and 4- hydroxyphenylacetaldehyde (Figure 1). The four identified NCSs (NNU14334, NNU21730, NNU21731, and NNU21732) were classified into two groups (Figure 6A). NNU21730 is closely related to *Populus euphratica, Theobroma cacao, Phoenix dactylifera*, and *Elaeis guineensis*. Meanwhile, the other three NCSs are closely related to *T. flavum, C. Japonica, Corydalis saxicola, P. somniferum*, and *Argemone mexicana*. The expression of NNU21730 for the four stages in two cultivars differed from those of the other three genes (Figures 6B–E). NNU21730 was up-regulated as the stages progressed, and was expressed more highly in “10–48” than in “Luming Lian.” The expression of NNU14334 and NNU21731 increased at stages S1 and S2, and then decreased from stage S3. NNU21732 had a low level of expression, which may have had a little effect on alkaloids. Considering the dynamic change of alkaloid content (Figure 2), NNU21730 was not specific for BIA biosynthesis, and NNU14334 exhibited phenotypic lag, wherein its expression was prior to the dynamic phenotypic changes of alkaloid content.

**Figure 7:**
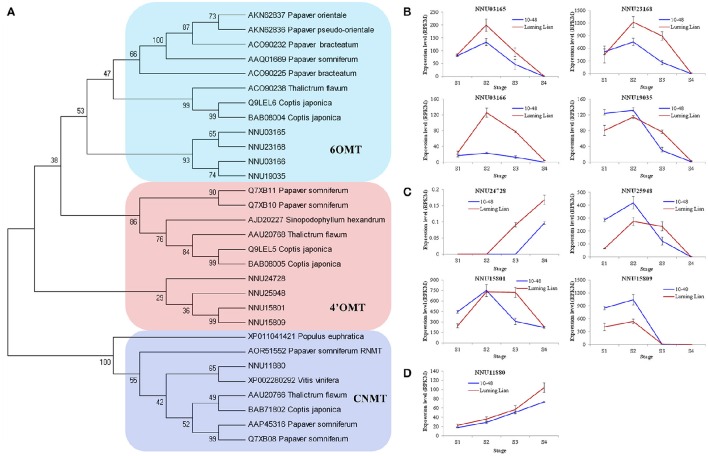
**Phylogenetic relationships of the genes encoding three methyltransferases in the BIA pathways for different species based on protein sequence (A)** and the expression of these genes at four developmental stages in two lotus cultivars **(B–D)**. The three methyltransferases are 6-O-methyltransferase (6OMT), 3′-hydroxy-N-methylcoclaurine 4′-Omethyltransferase (4′OMT), and coclaurine N-methyltransferase (CNMT). In **(A)**, the value at each node is the bootstrap support value from 1000 replicates. In **(B–D)**, RPKM value for each gene used three biological replicates; the error bars indicate standard error (*n* = 3).

There are two O-methyltransferases (6OMT and 4′OMT) and an N-methyltransferase (CNMT) in the common pathway of the BIA biosynthesis (Figure 1). The genes encoding these enzymes were identified (Figure 7A). The four homologs encoding 6OMT (NNU03165, NNU03166, NNU19035, and NNU23168) had the highest expression at stage S2, and their expression in “Luming Lian” was significantly higher than that in “10–48” (Figure 7B). Of the four homologs encoding 4′OMT, NNU24728 had low expression, although it was up-regulated as the stages progressed. The other three homologs, NNU15801, NNU15809, and NNU25948, had the highest expression at stage S2, and their expression in “10–48” was significantly higher than that in “Luming Lian” in the first three stages (Figure 7C). The homolog encoding CNMT, NNU11880, was up-regulated as the stages progressed, and its expression in “Luming Lian” was significantly higher than that in “10–48” (Figure 7D). Considering the dynamic change in alkaloid content (Figure 2), the three homologs of 6OMT (NNU03165, NNU03166, and NNU23168) exhibited phenotypic lag, wherein their expression were prior to the dynamic phenotypic changes of alkaloid content. CNMT is positively correlated with alkaloid accumulation.

**Figure 8:**
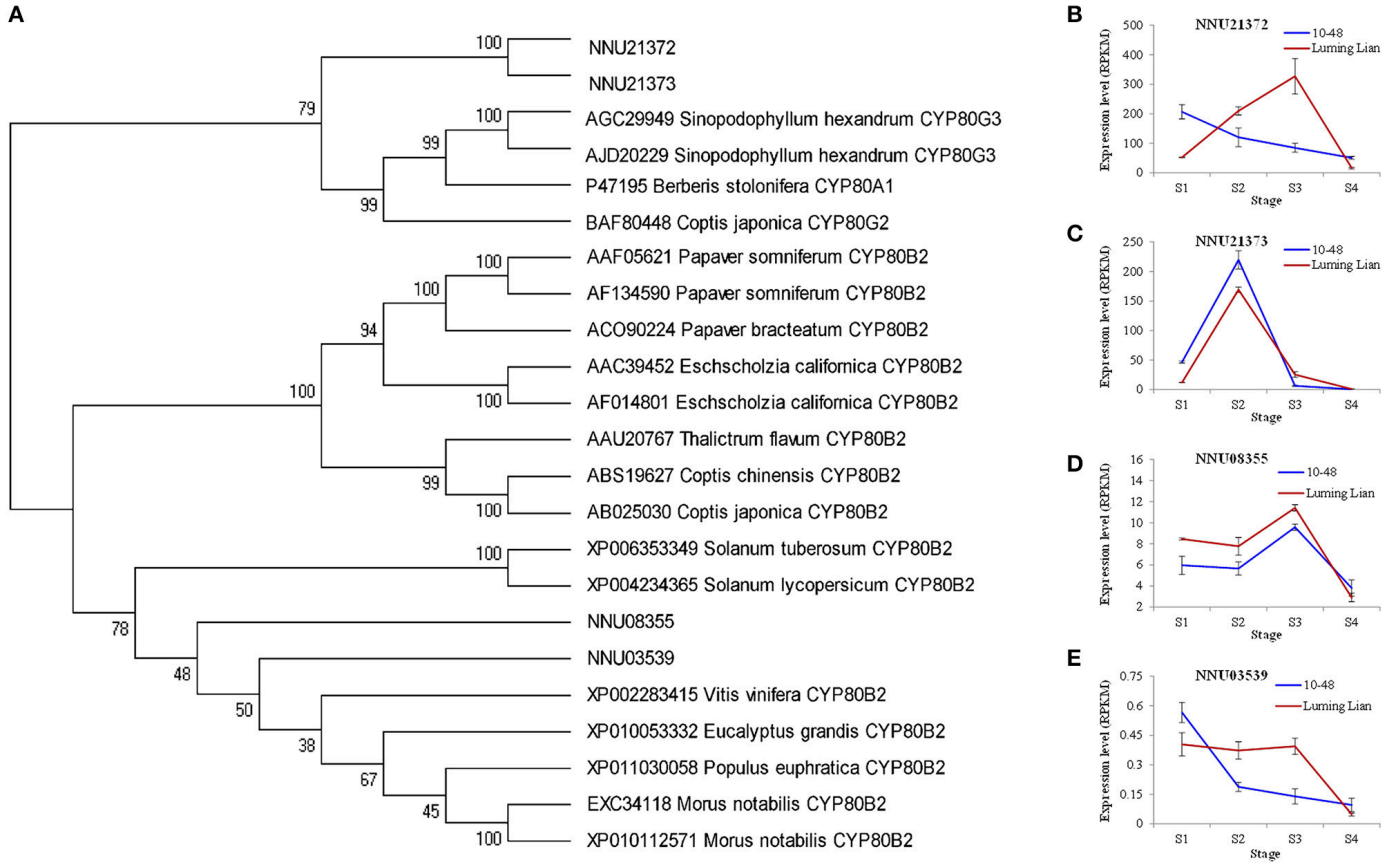
**Phylogenetic relationships of NMCH (***CYP80B***) and CTS (***CYP80G***) for different species based on protein sequence (A)** and the expression of these genes at four stages in two lotus cultivars **(B–E)**. In **(A)**, the value at each node is the bootstrap support value from 1000 replicates. In **(B–E)**, RPKM value for each gene used three biological replicates; the error bars indicate standard error (*n* = 3).

CYP80B and CYP80G encode NMCH and CTS, the committed enzymes for the common pathway and aporphine alkaloids branch, respectively (Figure 1). Two homologs for CYP80B (NNU03539 and NNU08355) and two homologs for CYP80G (NNU21372 and NNU21373) were identified (Figure 8A). The expression of NNU03539 was low in the two cultivars, so it may have had little effect on alkaloids (Figure 8E). The expression of NNU08355 peaked at stage S3 in the two cultivars, which was significantly higher in “Luming Lian” than in “10–48” (Figure 8D). In addition, the expression pattern of NNU21372 differed between the two cultivars. In “10–48” it was down-regulated as the stages progressed, while in “Luming Lian,” it peaked at stage S3. The expression level of NNU21372 in “Luming Lian” was higher than that in “10–48” at stages S2 and S3 (Figure 8B). The expression of NNU21373 peaked at stage S2 in the two cultivars, and was significantly higher in “10–48” than in “Luming Lian” (Figure 8C). Combining these findings with the dynamic change of alkaloid content (Figure 2), we predicted that CYP80B (NNU08355) and CYP80G (NNU21372) would have positive effects on alkaloid content, albeit with phenotypic lag.

The homologs encoding SOMT, CODM, and T6ODM were identified (Supplementary Figure 2). There were three homologs (NNU20253, NNU20903, and NNU24927) encoding SOMT, which is involved in the protoberberine alkaloid pathway. The expression of NNU20253 in “Luming Lian” were significantly higher than that in “10–48” as the developmental stages progressed. NNU20903 only showed significant difference between the two cultivars at stage S1. The expression of NNU24927 increased as the stages progressed, and was significantly higher in “Luming Lian” than in “10–48” (Supplementary Figure 2A). One gene encoding CODM (NNU26506) and two genes encoding T6ODM (NNU14369 and NNU16999) were identified that are involved in the morphinan alkaloid pathway. NNU26506 was significantly up-regulated as the stages progressed, but showed no significant difference between the two cultivars (Supplementary Figure 2B). NNU14369 only showed a significant difference between the two cultivars at stage S1. The expression of NNU16999 increased as the developmental stages progressed, and was significantly higher in “Luming Lian” than in “10–48” (Supplementary Figure 2C).

### Genes potentially involved in the late steps of aporphine alkaloid biosynthesis predicted by WGCNA

The five predominant alkaloids in lotus are aporphine alkaloids. The first two steps in aporphine alkaloid biosynthesis are to yield corytuberine and magnoflorine. Little is known about the late steps how corytuberine and magnoflorine produce these five aporphine alkaloids of lotus. Given the structural analysis of the five aporphine alkaloids, we conjectured that oxidoreductases and methyltransferases are involved in the late steps. Therefore, we used WGCNA to predict the genes involved in the late steps of aporphine alkaloid biosynthesis.

**Figure 9:**
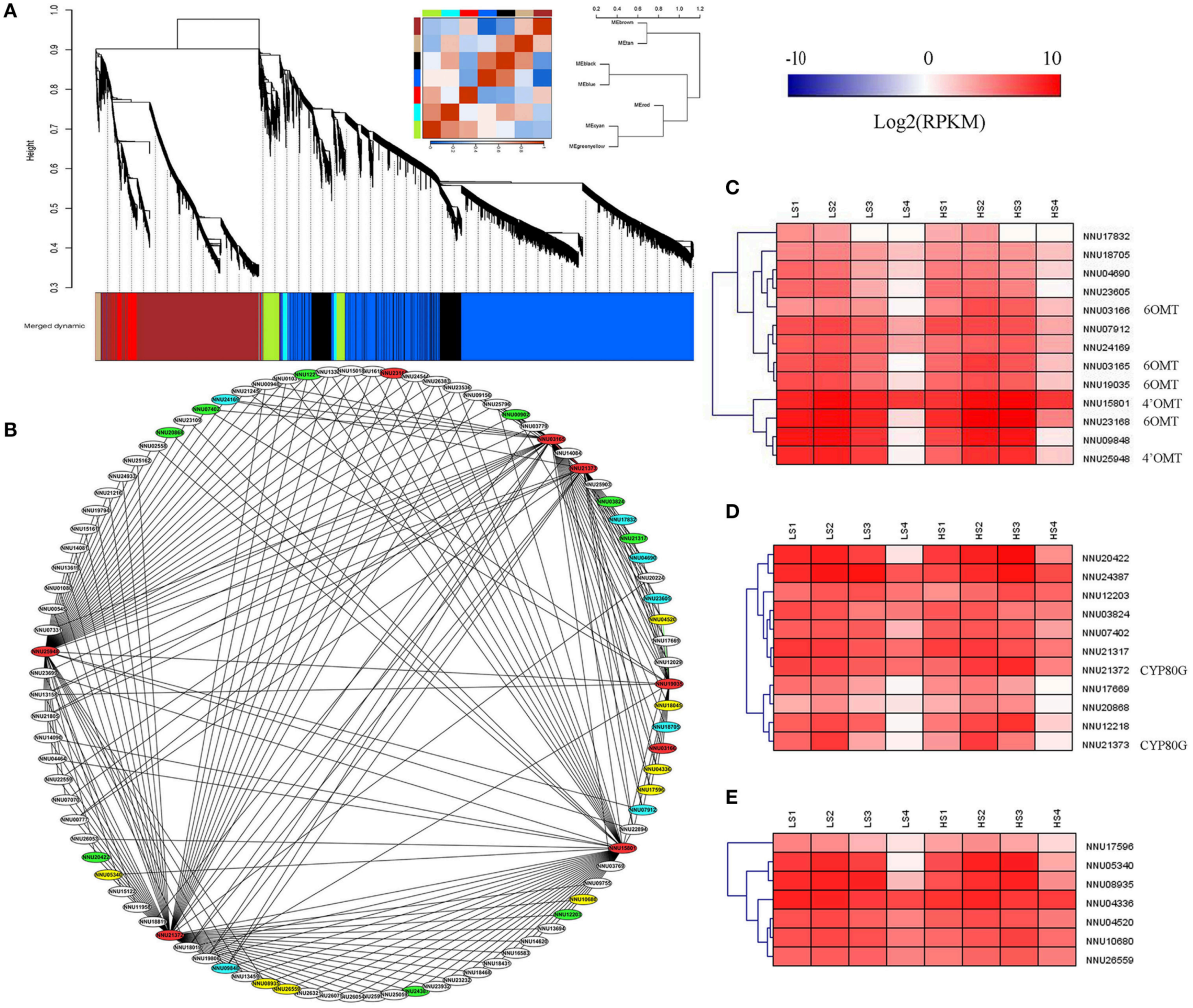
**Weighted gene co-expression network analysis (WGCNA) of differentially expressed genes (DEGs) identified from the two lotus cultivars at four developmental stages. (A)** Hierarchical cluster tree showing seven modules of co-expressed genes. Each of the DEGs is represented by a leaf in the tree. Seven modules were identified, which are shown in designated colors: “Black,” “Blue,” “Brown,” “Cyan,” “Greeenyellow,” “Red,” and “Tan.” **(B)** Cytoscape representation of co-expressed genes with edge weight ≥0.15 in module “Greenyellow.” Gene IDs in red color refer to 6OMT, 4′OMT, and CYP80G, the ones in blue refer to methyltransferases, the ones in green refer to CYP450, and the ones in yellow refer to oxidoreductases. The other genes are in white. **(C)** Heatmap of methyltransferases across the four developmental stages in the lotus cultivars, “10–48” and “Luming Lian.” **(D)** Heatmap of CYP450 across the four developmental stages in the lotus cultivars, “10–48” and “Luming Lian.” **(E)** Heatmap of oxidoreductases across the four developmental stages in the lotus cultivars “10–48” and “Luming Lian.” Expression values are presented as RPKM normalized log_2_transformed counts. Red and blue colors indicate up- and down- regulated transcripts, respectively.

WGCNA was performed with all DEGs, which enabled the identification of seven WGCNA modules (Figure 9A). The module “Greenyellow” had expression patterns that were associated with the dynamic change of alkaloid content. Ninety genes with WGCNA edge weight >0.15 were selected in this module, which indicated that these genes were highly connected among them. As a result, 329 pairs of co-expression edges were linked among these genes (Figure 9B). Among the 90 genes, 13 were methyltransferases, 11 were CYP450 family genes, eight were oxidoreductases, 30 encoded the enzymes with other catalytic activity, 19 encoded uncharacterized proteins, and the others encoded transporters or transcription factors. For the 13 methyltransferases, six were 6OMT and 4′OMT. For the 11 genes for CYP450, two were CYP80G, which encodes CTS (Figures 9B–D and Supplementary Table 4).

DGE analysis showed that the seven methyltransferase genes had the highest expression at stage S2 or S3, which was similar to the expression patterns of 6OMT and 4'OMT (Figure 9C). For the CYP450s, six and three genes had patterns similar to the CYP80Gs, NNU21372, and NU21373, respectively (Figure 9D). The eight oxidoreductase genes were expressed most highly at stage S2, and “Luming Lian” had higher expression than “10–48” in the last three stages (Figure 9E). Eleven genes were found to cluster closely with 6OMT, 4′OMT, and CYP80G (correlation >0.96), including five methyltransferase genes (NNU04690, NNU07912, NNU09848, NNU23605, and NNU24169), one CYP450 gene (NNU21317), and five oxidoreductase genes (NNU04336, NNU04520, NNU05340, NNU08935, and NNU10680). These 11 genes were conjectured to be involved in the late steps of aporphine alkaloid biosynthesis.

### Experimental confirmation of gene expression by RT-qPCR

**Figure 10:**
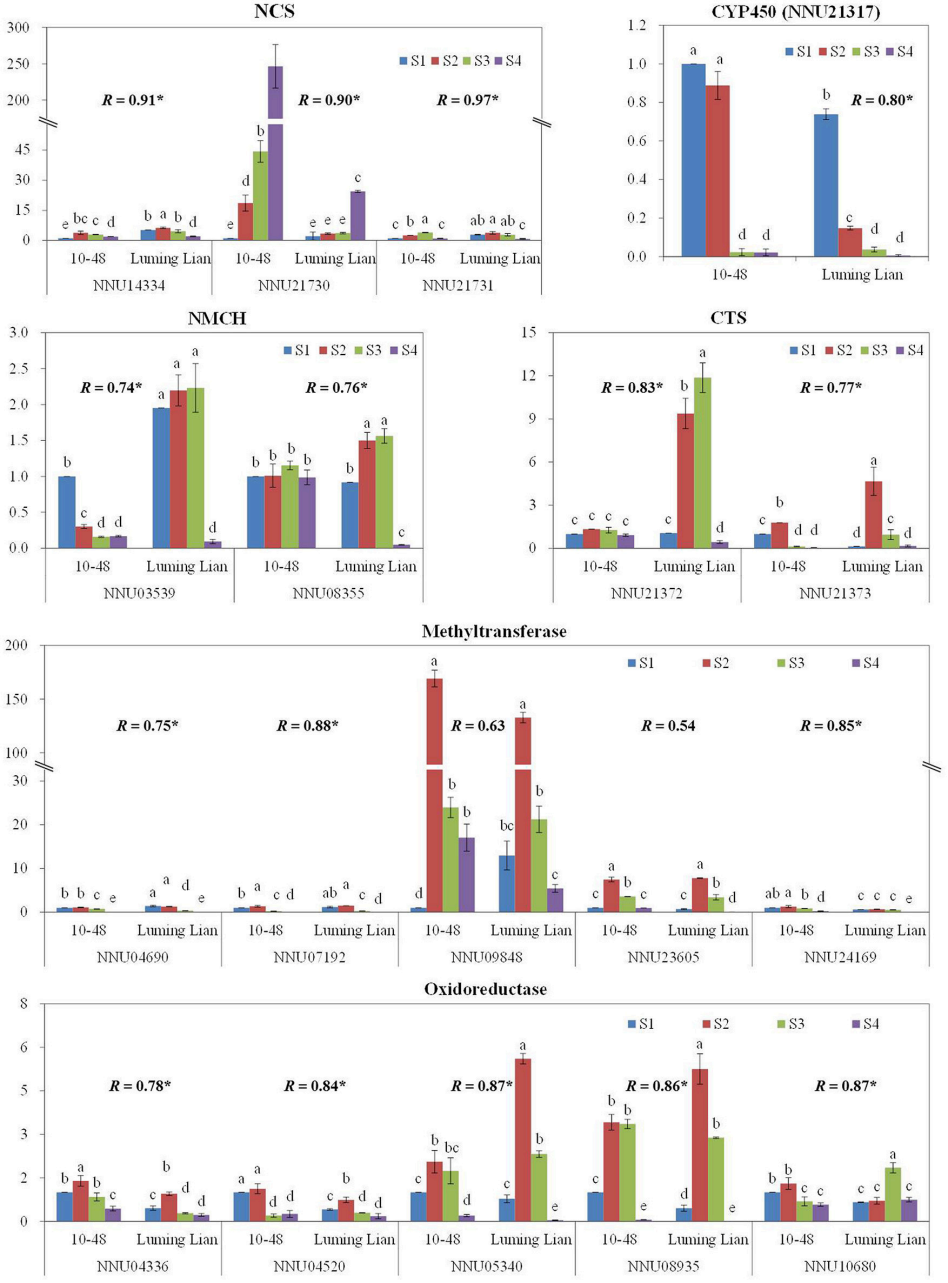
**Quantitative real-time RT-qPCR confirmation of seven genes at the four stages, S1, S2, S3, and S4, between the two cultivars, “10–84” and “Luming Lian.”** The left y axis indicates relative gene expression levels determined by RT-qPCR. Relative gene expression was normalized by comparison with the expression of lotus β actin (NNU24864). The expression values were adjusted by setting the expression of LS1 to 1 for each gene. All RT-qPCRs for each gene used three biological replicates, with three technical replicates per experiment; the error bars indicate standard error. Different lower-case letters (a–e) indicate a significant difference among eight treatments at *P* = 0.05. Correlation coefficient (*R*) shown for each gene between the RT-qPCR and DGE data are those that reached significance at *P* < 0.05.

To confirm the results obtained through DGE analysis, 18 genes involved in the pathways of BIA biosynthesis were selected to analyze their expression in a biologically independent experiment by RT-qPCR, including three genes encoding NCS, two encoding NMCH, two genes encoding CTS, and the 11 candidate genes (Figure 10). The RT-qPCR results for all of the genes were tested statistically, and each gene showed significantly different expression among the treatments (*P* = 0.05). Moreover, 16 genes, except for NNU09848 and NNU23605, showed significant correlations (*P* = 0.05) between the RT-qPCR data and the RNA-seq results, which indicated that the expression of the genes studied was consistent between the RT-qPCR and DGE analyses, despite some quantitative differences in expression level (Figure 10).

## Discussion

### The importance of alkaloid in *Nelumbo nucifera*

Alkaloids are the most important active components in lotus leaves, playing important roles in treating various diseases (Lacour et al., 1995; Kashiwada et al., 2005; Ono et al., 2006; Huang et al., 2010; Nguyen et al., 2012; Poornima et al., 2014). Alkaloids were present in each organ of lotus, such as leaf, petiole, petal, seed, plumule and rhizome (Chen et al., 2013b; Zhou et al., 2013; Deng et al., 2016). Lotus leaves and plumules are rich in BIAs, whereas petioles and rhizomes contain trace amounts of alkaloids. In leaves, five aporphine-type alkaloids, N-nornuciferine, O-nornuciferine, anonaine, nuciferine, and roemerine are the most dominant component. Alkaloid composition and content ranged from different lotus genotypes (Chen et al., 2013b; Deng et al., 2016). These five aporphine alkaloids were identified at the six developmental stages of leaves in two lotus cultivars. Nuciferine was the dominant alkaloid in both cultivars, followed by N-nornuciferine in “10–48” and O-nornuciferine in “Luming Lian,” respectively (Figure 2). This result was consistent with a previous study (Deng et al., 2016). Total content and the dynamic change of alkaloids in two cultivars was different. Alkaloid content in “Luming Lian” increased rapidly throughout the period, and was 4.6 times higher than that of “10–48” at last stage (Figures 2C,D), which was same as the result tested by Deng et al. (2016). Hence, “10–48” and “Luming Lian” are the ideal materials to study aporphine alkaloids in lotus.

Because of the pharmacological significance of lotus, more and more researches on lotus alkaloid have been reported, including the pharmacological function of alkaloid, the qualitative and quantitative analysis of alkaloid composition, the alkaloid variation among lotus genotypes, and so on (Ono et al., 2006; Huang et al., 2010; Nguyen et al., 2012; Chen et al., 2013b; Poornima et al., 2014; Deng et al., 2016). Lotus NCS genes were isolated and characterized (Vimolmangkang et al., 2016), however, less report is on aporphine alkaloid biosynthesis in lotus. Among the different BIAs, only the details of the aporphine alkaloid biosynthetic pathway remain largely unclear, while the biosynthetic pathways for the other alkaloids have been well elucidated (Liscombe and Facchini, 2008; Ziegler and Facchini, 2008; Chow and Sato, 2013; Glenn et al., 2013; Hagel and Facchini, 2013). In order to characterize the aporphine alkaloid pathway in lotus, four stages of leaf development that significantly differ between “10–48” and “Luming Lian” were selected to detect genes expression using DGE technology (Figure 2).

Ninety-four percent of clean pair-end reads were aligned against the lotus “China Antique” reference genome, and 86% of the predicted genes in the lotus genome were identified (Ming et al., 2013), which is consistent with previous estimates of the flower and rhizome transcriptome generated in lotus by RNA-seq studies (Yang et al., 2014, 2015). Dozens of DEGs were assigned to the categories of biosynthesis of secondary metabolites, isoquinoline alkaloid biosynthesis, and flavonoid biosynthesis by KEGG analysis (Figure 5). This provided a meaningful framework for the specific biological activities. The secondary metabolisms vary considerably depending on the enzyme(s) associated with the pathways. Lotus contain abundant alkaloids, steroids, flavonoids, triterpenoids, glycosides and polyphenols (Chen et al., 2013a,b; Deng et al., 2013, 2016). The expressions of the genes in biosynthesis of secondary metabolites were changed with the development of the lotus leaf, and regulated the content of these metabolites. Lotus aporphine alkaloids are isoquinoline alkaloid, and the genes involved in isoquinoline alkaloid pathway should differentially express. The KEGG result confirmed the accuracy of the data and provided new clues that deepen the understanding of aporphine alkaloids metabolism in lotus.

### Genes related to BIA biosynthesis in lotus

BIAs constitute the group of the most abundant alkaloids in the plant. They are derived biosynthetically from the amino acid tyrosine generating the fundamental intermediate dopamine and 4-hydroxyphenyl acetaldehyde, and yielded through the branch pathways, including morphinan, benzophenanthridine, protoberberine and aporphine alkaloid pathways (Liscombe and Facchini, 2008; Ziegler and Facchini, 2008; Chow and Sato, 2013; Glenn et al., 2013; Hagel and Facchini, 2013). The genes for the common pathway and the branches (morphinan, benzophenanthridine and protoberberine) have been cloned in the opium poppy, Japanese goldthread, and yellow meadow rue. The two enzymes in aporphine alkaloid pathway, CTS and RNMT, were characterized in Japanese goldthread and opium poppy, respectively (Kraus and Kutchan, 1995; Ikezawa et al., 2008; Liscombe and Facchini, 2008; Ziegler and Facchini, 2008; Chow and Sato, 2013; Glenn et al., 2013; Morris and Facchini, 2016). In lotus, the genes involved in the common pathway and aporphine alkaloid pathway were identified, which were NCS, 6OMT, CNMT, NMCH, 4′OMT and CTS (Figures 6–8).

NCS is the first committed enzyme in the common pathway, and catalyzes the condensation of dopamine and 4-hydroxyphenyl acetaldehyde to (*S*)-norcoclaurine. It plays an important role in the regulation of BIA biosynthesis due to its entry-point location in the pathway (Minami et al., 2007; Liscombe and Facchini, 2008; Ziegler and Facchini, 2008; Chow and Sato, 2013; Glenn et al., 2013). In plant, the NCS genes are divided into two subfamilies. NCSI genes are only identified in a limited number of dicotyledonous taxa that produce BIAs, while NCSII genes are universal in plants (Lee and Facchini, 2010). Seven NCS genes have been identified in the sacred lotus genome. The high or low levels of alkaloids may be inhibited or induced, respectively, by the expression of NnNCS7, one of NCSI (Vimolmangkang et al., 2016). Four of these seven NCS genes were also detected in our study. The expression of NNU21730, belongs to NCSII, was not significantly correlated with alkaloid content in leaf (Figures 6A,B). It is not specific to BIA biosynthesis and its lack of expression may not inhibit alkaloid accumulation (Vimolmangkang et al., 2016). NNU21732, the NCSI gene, had a low expression level (Figure 6D) and is a pseudogene because of the single-nucleotide deletion adjacent to the GC dinucleotide at the 5′ border of the intron (Vimolmangkang et al., 2016). The dynamic phenotypic change of alkaloid content occurs later than the actual expression change of NNU14334 and NNU21731, namely phenotypic lag (Figures 6C,E). None of these four identified genes showed a significant correlation with alkaloid content in leaves in this study. It is likely that other NCS homologs, for example, *NnNCS7*, play a prominent role in alkaloid accumulation in leaves (Vimolmangkang et al., 2016). More studies are needed to confirm the function of NCS in the biosynthesis of BIAs.

The conversion of (*S*)-norcoclaurine to (*S*)-reticuline involves a series of enzymes: 6OMT, CNMT, NMCH, and 4′OMT (Liscombe and Facchini, 2008; Ziegler and Facchini, 2008; Chow and Sato, 2013; Glenn et al., 2013). Among them, 6OMT, CNMT, and 4′OMT are methyltransferases. Specifically, 6OMT and 4′OMT are both class II O-methyltransferases and display strict regiospecificity (Ziegler et al., 2005). 6OMT is the rate-limiting gene, and 4′OMT is a surrogate for 6OMT that facilitates BIA biosynthesis. Constitutive overexpression of 6OMT led to increased alkaloid content. In contrast, overexpression of 4′OMT was shown to have little effect on alkaloid content (Inui et al., 2007; Desgagné-Penix and Facchini, 2012). Four homologs for each of 6OMT and 4′OMT were found in lotus, and were clustered with taxa that produce BIAs, such as *P. somniferum, T. flavum*, and *C. japonica* (Figure 7A). 6OMT showed differential expression pattern with 4′OMT in “Luming Lian” and “10–48” (Figures 7B,C). 6OMT had positive regulation on alkaloid content, while 4′OMT inhibited alkaloid accumulation, which confirmed their distinct effects on alkaloid regulation. Furthermore, their expressions were prior to the dynamic changes of aporphine alkaloid content. This phenomenon of phenotypic lag may be due to that 6OMT and 4′OMT are not specific to aporphine alkaloid biosynthesis. NMCH is encoded by *CYP80B*, a gene in P450 superfamily and is highly region- and stereo-specific (Liscombe and Facchini, 2008; Ziegler and Facchini, 2008; Chow and Sato, 2013; Glenn et al., 2013). Overexpression of *CYP80B* resulted in an increase in the amount of total alkaloid in latex of opium poppy, but without changing the ratio of the individual morphine alkaloids (Pauli and Kutchan, 1998; Frick et al., 2007). VIGS-based silencing of *CYP80B* did not affect papaverine levels (Desgagné-Penix and Facchini, 2012). The expression of *CYP80B*, NNU08355, has a positive effect on aporphine alkaloids content in lotus, but exhibits phenotypic lag (Figures 8, 10), which might be because of NMCH which acts early in the BIA biosynthetic pathway (Frick et al., 2007).

In the aporphine alkaloid pathway, CTS, the first committed enzyme, has intramolecular C–C phenol-coupling activity to produce corytuberine from (*S*)-reticuline. It is encoded by *CYP80G*, and was firstly cloned and characterized in Japanese goldthread (Ikezawa et al., 2008). *CYP80G* was shown to be similar in respect of high amino acid sequence to *CYP80A*, a berbamunine synthase involved in the bisbenzylisoquinoline alkaloid pathway (Ikezawa et al., 2008; Ziegler and Facchini, 2008). Two homologs of *CYP80G* found in our study exhibited different expression patterns (Figures 8, 10). Combining their expression pattern with the dynamic change of alkaloid content, we predicted that NNU21372 has a positive effect on aporphine alkaloid content, but NNU21373 has no specific effect on this variable. The gene RNMT is responsible for the yield of magnoflorine from corytuberine, and was isolated in *P. somniferum*. Suppression of RNMT resulted in a significant decrease in magnoflorine accumulation, and a concomitant increase in corytuberine levels (Morris and Facchini, 2016). RNMT is a member of NMTs, and forms a larger clade with CNMTs from *T. flavum, C. japonica*, and *P. somniferum*, which is in agreement with our analysis (Figure 7A). However, its homolog was not found in lotus.

Transcription factors, *WRKY1* and *bHLH1*, regulated the BIA metabolism. The expression of the two transcription factors caused a substantial increase in the expression of several berberine biosynthetic genes (Kato et al., 2007; Yamada et al., 2011). Transcription factors may be used to improve the yields of BIAs. To test whether these two transcription factors regulate the BIAs biosynthesis in lotus, we selected *WRKY* and *bHLH* which differentially expressed at the four developmental stages in the two cultivars (Supplementary Figure 3). 35 *WRKY* and 45 *bHLH1* were identified. Some had similar expression tendency as the dynamic change of alkaloid content. However, no one was predicted using WGCNA. Further studies are needed to test the function of *WRKY1* and *bHLH1* in regulating the BIAs biosynthesis.

As stated above, we concluded that these homologous genes, NCS (NNU14334), 6OMT (NNU03165, NNU03166, and NNU23168), CNMT (NNU11880), NMCH (NNU08355), 4′OMT (NNU15801 and NNU25948), and CTS (NNU21372), are involved in the common pathway and aporphine alkaloid pathway in lotus. They are thus candidate genes for alkaloid biosynthesis.

### Candidate genes for the late steps of the aporphine alkaloid pathway

WGCNA can capture the relationships of individual genes comprehensively, and is a powerful tool for obtaining new insights into both the function of genes and the mechanism controlling complex traits (Fuller et al., 2011; Wisniewski et al., 2013). WGCNA was developed to analyze more efficiently microarray datasets and transcriptomic profiling experiments. This method has been used successfully to dissect fruit anthocyanin in apple (*Malus* × *domestica*) (El-Sharkawy et al., 2015), the tomato fruit metabolome (DiLeo et al., 2011; Gao et al., 2013), and pollination in petunias (Broderick et al., 2014). In this study, WGCNA was used to predict genes that may be involved in the aporphine alkaloid pathway of lotus.

Upon performing structural comparisons corytuberine and magnoflorine with five prominent aporphine alkaloids in lotus, we predicted that these aporphine alkaloids were produced through the chemical reactions of oxidation, dehydroxylation, methyl transfer, and demethylation (Figure 1). The enzymes involved in the late steps might be oxidoreductases and methyltransferases. CYP450 plays an important role in synthesizing plant secondary metabolites and has catalytic oxidation function for carbon–carbon bond, alkyl hydroxylation, and hydroxyl oxidation (Coon, 2005). Oxidoreductases involved in aporphine alkaloid biosynthesis are likely to come from the CYP450 family. WGCNA identify 90 genes associated with alkaloid content, and 32 genes were methyltransferases, CYP450 genes, and oxidoreductases (Figures 9A,B and Supplementary Table 4). In the biosyntheses of BIAs, CYP450-mediated hydroxylation, methylenedioxy bridge formation, and phenolcoupling reactions have been reported. CYP80A, CYP80B, and CYP80G catalyze hydroxylation and C–O phenol-coupling, and CYP719A catalyze methylenedioxy bridge formation. A series of reaction of BIA pathways were catalyzed by methyltransferases and oxidoreductases (Chow and Sato, 2013; Glenn et al., 2013; Hagel and Facchini, 2013). Among the 32 predicted genes, eight were 6OMT, 4′OMT, and CYP80G, and the remaining genes are likely to be involved in the late steps of aporphine alkaloid biosynthesis (Figures 9C–E).

6OMT, 4′OMT, and CYP80G are key enzymes in the common pathway of BIA synthesis and the aporphine alkaloid branch, and their expression affects the metabolic activities of downstream factors. Therefore, the expression profiles of the candidate gene in the late steps of aporphine alkaloid synthesis should match the expression profiles of 6OMT, 4′OMT, and CYP80G. Eventually, 11 candidate genes were identified for the late steps of the aporphine alkaloid pathway. The expression of these candidate genes was confirmed by RT-qPCR (Figure 10). Since these genes had not been characterized previously, this study provides not only new insights into the aporphine alkaloid pathway, but also a list of interesting candidate genes for more dedicated functional studies in the future. Functional analysis of these candidate genes might be useful for genetic engineering to regulate aporphine alkaloid content in lotus.

## Author contributions

MY participated in the design of the study, analyzed the data, and drafted the manuscript. LZ collected the leaves samples, extracted RNA, and sequenced the RNA libraries. LL aligned the sequenced data to the reference genome and performed gene expression analysis. JL conducted Gene Ontology enrichment and KEGG analysis. LX cultivated and provided the plant materials. JF constructed gene co-expression network and predicted the candidate genes. YL conceived the study, participated in its design and coordination, and helped to draft the manuscript. All authors read and approved the final manuscript.

### Conflict of interest statement

The authors declare that the research was conducted in the absence of any commercial or financial relationships that could be construed as a potential conflict of interest.

## Supplementary material

Supplementary Figure 1 **Number of genes expressed at the four stages in the two cultivars. (A)** Venn diagram of genes identified in “10–48” (low-alkaloid cv.) and “Luming Lian” (high-alkaloid cv.). **(B)** Venn diagram of genes in “10–48” (low-alkaloid cv.) at four developmental stages. **(C)** Venn diagram of genes in “Luming Lian” (high-alkaloid cv.) at four developmental stages. The individual and overlapping areas in Venn diagrams represent the numbers of specifically expressed and co-expressed genes between the different stages, respectively.

Supplementary Figure 2 **The expression of the genes encoding scoulerine-9-O-methyltransferase (SOMT), codeine 3-O-demethylase (CODM), and thebaine 6-O-demethylase (T6ODM) at four developmental stages in the two cultivars**. RPKM value for each gene used three biological replicates; the error bars indicate standard error (*n* = 3).

Supplementary Figure 3 **Heatmap of transcription factors, ***WRKY*** (A)** and *bHLH1* **(B)** across the four developmental stages in the lotus cultivars “10–48” and “Luming Lian.” Expression values are presented as RPKM normalized log_2_transformed counts.

Supplementary Table 1 **Primers used to assay gene expression by RT-qPCR**.

Supplementary Table 2 **Summary statistics of clean reads in the transcriptomes of lotus**.

Supplementary Table 3 **Pearson's correlation coefficients for three biological replicates at each stage in the two cultivars**.

Supplementary Table 4 **Expression of the genes in the co-expression network**.
