# Supplementary figures and images for: Digital Gene Expression Analysis Provides Insight into the Transcript Profile of the Genes Involved in Aporphine Alkaloid Biosynthesis in Lotus (Nelumbo nucifera)
Source: Front Plant Sci. 2017 Jan 31;8:80. doi: 10.3389/fpls.2017.00080 (PMC5281601; doi:10.3389/fpls.2017.00080)

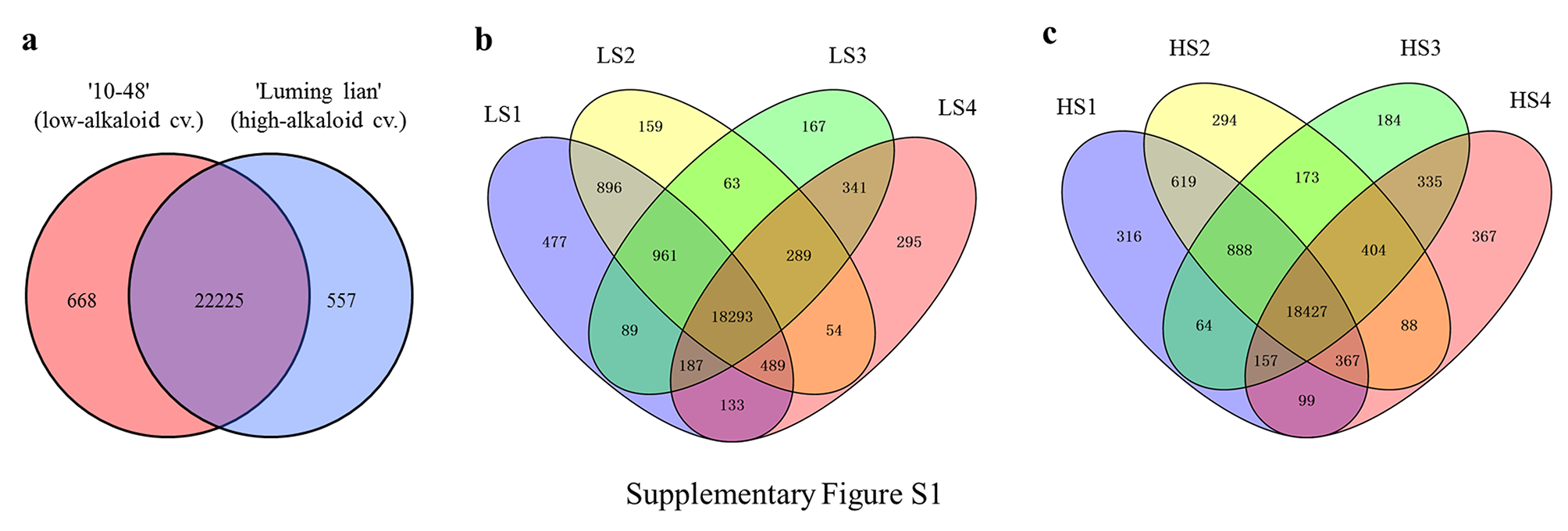

Supplement: Supplementary Figure 1 — Number of genes expressed at the four stages in the two cultivars. (A) Venn diagram of genes identified in “10–48” (low-alkaloid cv.) and “Luming Lian” (high-alkaloid cv.). (B) Venn diagram of genes in “10–48” (low-alkaloid cv.) at four developmental stages. (C) Venn diagram of genes in “Luming Lian” (high-alkaloid cv.) at four developmental stages. The individual and overlapping areas in Venn diagrams represent the numbers of specifically expressed and co-expressed genes between the different stages, respectively. [file Image1.TIF]

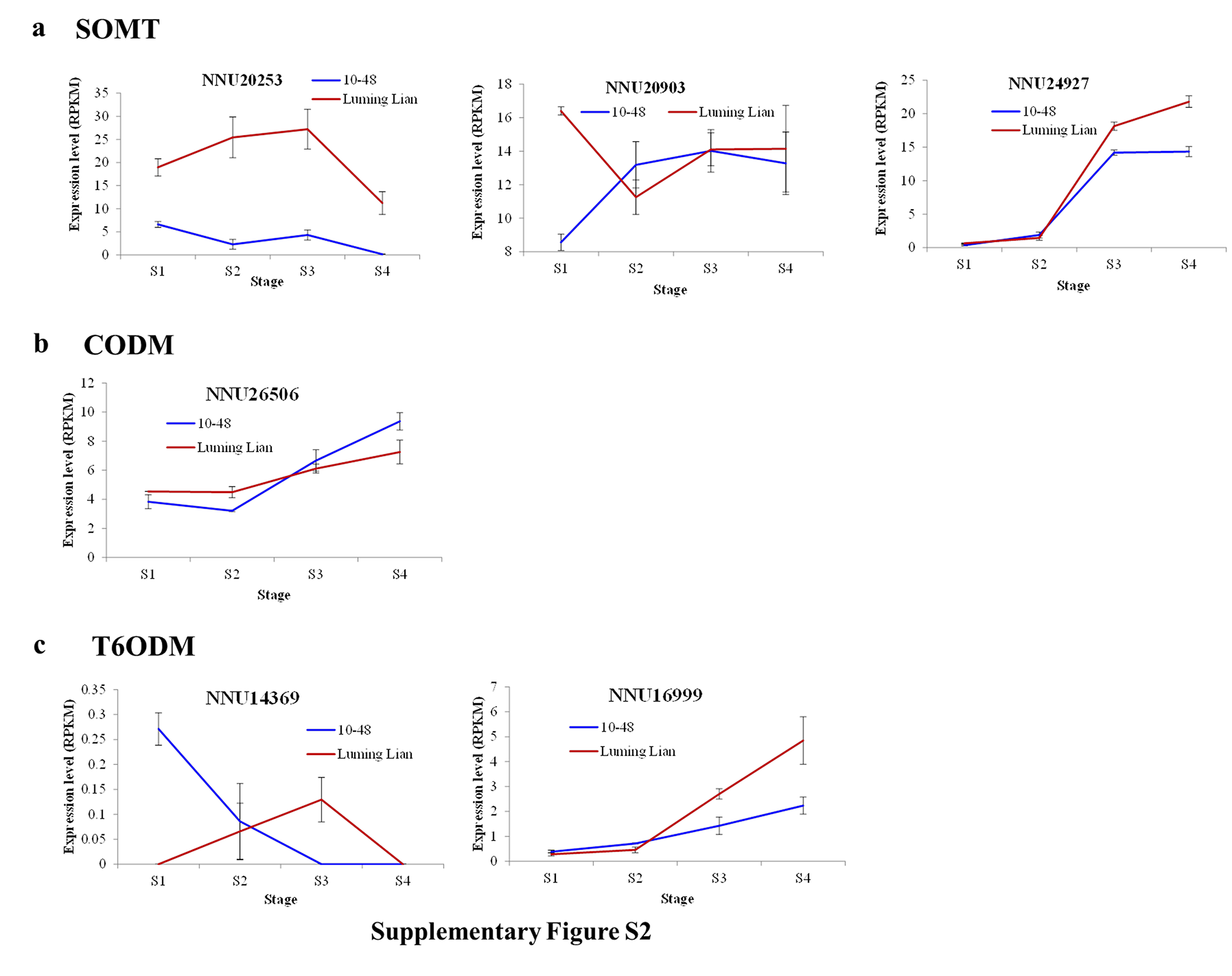

Supplement: Supplementary Figure 2 — The expression of the genes encoding scoulerine-9-O-methyltransferase (SOMT), codeine 3-O-demethylase (CODM), and thebaine 6-O-demethylase (T6ODM) at four developmental stages in the two cultivars. RPKM value for each gene used three biological replicates; the error bars indicate standard error (n = 3). [file Image2.TIF]

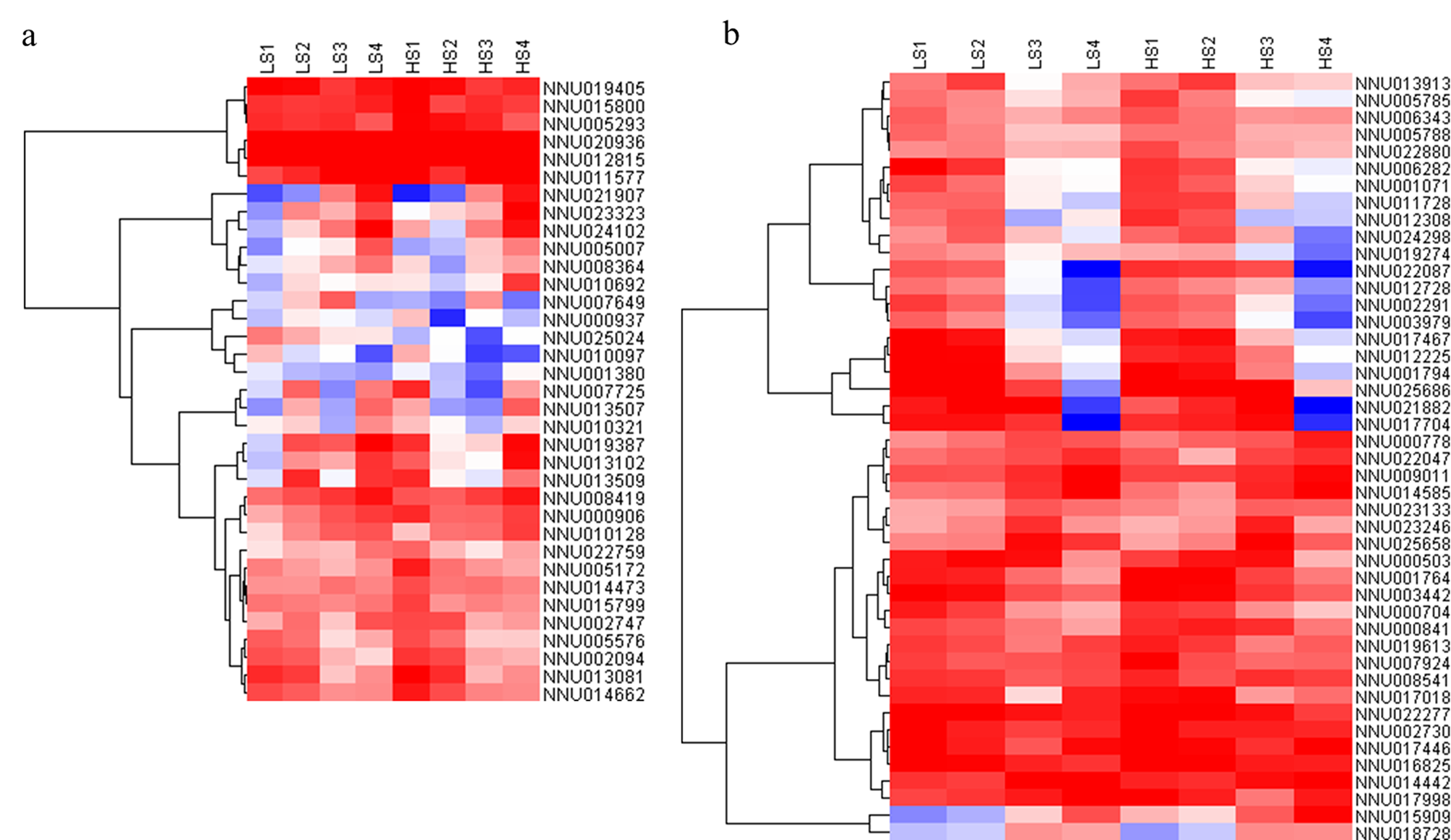

Supplement: Supplementary Figure 3 — Heatmap of transcription factors, WRKY (A) and bHLH1 (B) across the four developmental stages in the lotus cultivars “10–48” and “Luming Lian.” Expression values are presented as RPKM normalized log2transformed counts. [file Image3.TIF]
