# Supplementary material for: Digital Gene Expression Analysis Provides Insight into the Transcript Profile of the Genes Involved in Aporphine Alkaloid Biosynthesis in Lotus (Nelumbo nucifera)
Source: Front Plant Sci. 2017 Jan 31;8:80. doi: 10.3389/fpls.2017.00080 (PMC5281601; doi:10.3389/fpls.2017.00080)
Supplement: Supplementary Table 1 — Primers used to assay gene expression by RT-qPCR. [file Table1.DOCX]

**Supplementary Table 1** Primers used to assay gene expression by RT-qPCR

| Gene ID | Gene name | Forward primer (5’→3’) | Reverse primer (5’→3’) |
| --- | --- | --- | --- |
| NNU14334 | *NCS* | ACGAATGAATTAGATGTGGACG | ACGATGTGGGTGGGTAGCA |
| NNU21730 | *NCS* | GTCATCCGCAAGGCAGAA | ACGAAACCCAAGGTCCAA |
| NNU21731 | *NCS* | GCATCATTCGATCCACCAC | CGCTCCCCATAAATTACCAG |
| NNU08355 | *NMCH* | GCAAAGCCATCGAATCACA | TTCCCTTCCTTCCTACCCA |
| NNU03539 | *NMCH* | GGCAATGGCGGAGATGA | GAATGGCGCAGGAGGGT |
| NNU21372 | *CTS* | CTACCTTATCTCCATGCCTGTG | AAGTTTCCATTGCTCGGTGT |
| NNU21373 | *CTS* | CGACAGGGTAATCGGAAGA | AGGAATGGGACAGGAGGG |
| NNU24864 | *β-actin* | GATGCCCTGATGAAGATCC | CCACTCAGCACAATGTTTCC |
| NNU04690 |  | TGTGGAGATAACCAAGCAAAC | GGAGTATAATCAGTGTAACGAGCA |
| NNU07912 |  | GAGGAGTCCAAAACAGAAAATG | CTGTCTGCTCGCCTTCACTA |
| NNU09848 |  | TCAGGAGATAGCGGAGATGTAT | GGAGTTGGAACTTTTGGGTC |
| NNU23605 |  | GAAGTTATGAGGAAGTACAGGGAC | TGGGATAGTTCAATGCGAGA |
| NNU24169 |  | CTCGAAGCCCAGCATCTATA | GCAGTCTGTCTGTTTGGTAATCT |
| NNU21317 |  | GCTGTTGTCTGTCTCGCTTCT | GCAGGTTTTCCATCAATCTCA |
| NNU05340 |  | AATGGGGTTCAGACGGAGCA | AAGGGTCGGGGAGTTGGAAA |
| NNU04520 |  | GCAAGGGGTTATTGGGTGAC | ACAGAGGTGGGACTCGTGGG |
| NNU08935 |  | CGTTGCTACTCCAGTCCACTT | CCATTACGAGACCTGTTCCAG |
| NNU10680 |  | TGGTTATCCAGAGGCACATC | TTACAGCACCTACTTTCCTTTTC |
| NNU04336 |  | GCGAAGGTGAAGGAGACGAC | ATCAACACCTCCCGAGCATT |
