# Supplementary material for: Digital Gene Expression Analysis Provides Insight into the Transcript Profile of the Genes Involved in Aporphine Alkaloid Biosynthesis in Lotus (Nelumbo nucifera)
Source: Front Plant Sci. 2017 Jan 31;8:80. doi: 10.3389/fpls.2017.00080 (PMC5281601; doi:10.3389/fpls.2017.00080)
Supplement: Supplementary Table 2 — Summary statistics of clean reads in the transcriptomes of lotus. [file Table2.DOCX]

**Supplementary Table 2 Summary statistics of clean reads in the transcriptomes of lotus**

| Sample | Biological repeat | Raw reads | Clean reads | Total mapped | Q20 (%) | No. of expressed gene | No. of co-expressed gene |
| --- | --- | --- | --- | --- | --- | --- | --- |
| LS1 | R1 | 13992440 | 13910829 | 12978500 (93.3%) | 98.44 | 22514 | 21525 |
|  | R2 | 17370517 | 17292062 | 16145862 (93.4%) | 98.47 | 22813 |  |
|  | R3 | 15648038 | 15565372 | 14508160 (93.2%) | 98.46 | 22422 |  |
| LS2 | R1 | 13464422 | 13346017 | 12496848 (93.6%) | 98.63 | 22437 | 21204 |
|  | R2 | 12347094 | 12238198 | 11418858 (93.3%) | 98.62 | 22174 |  |
|  | R3 | 12605955 | 12429654 | 11622213 (93.5%) | 98.61 | 22405 |  |
| LS3 | R1 | 13343583 | 13228966 | 12424398 (93.9%) | 98.87 | 21757 | 20408 |
|  | R2 | 12558010 | 12465573 | 11694022 (93.8%) | 98.82 | 21489 |  |
|  | R3 | 14416139 | 14296965 | 13429036 (93.9%) | 98.89 | 21621 |  |
| LS4 | R1 | 12598866 | 12526240 | 11682082 (93.3%) | 98.87 | 21329 | 20081 |
|  | R2 | 12544427 | 12482556 | 11638557 (93.2%) | 98.88 | 21128 |  |
|  | R3 | 14026006 | 13976135 | 13088189 (93.7%) | 98.88 | 21597 |  |
| HS1 | R1 | 13268624 | 12900602 | 12194697 (94.5%) | 98.62 | 22339 | 20973 |
|  | R2 | 15686844 | 15084417 | 14265318 (94.6%) | 98.60 | 22615 |  |
|  | R3 | 14460662 | 14104143 | 13287260 (94.2%) | 98.61 | 21933 |  |
| HS2 | R1 | 14849758 | 14722314 | 13929700 (94.6%) | 98.87 | 22376 | 21260 |
|  | R2 | 14078977 | 13906287 | 13173817 (94.7%) | 98.86 | 22325 |  |
|  | R3 | 12756436 | 12593324 | 11919548 (94.6%) | 98.85 | 22235 |  |
| HS3 | R1 | 13563709 | 13137734 | 12392627 (94.3%) | 98.86 | 21831 | 20632 |
|  | R2 | 13334692 | 13248342 | 12523773 (94.5%) | 98.88 | 21594 |  |
|  | R3 | 15249326 | 14922359 | 14140387 (94.8%) | 98.88 | 21871 |  |
| HS4 | R1 | 16388428 | 16321335 | 15429621 (94.5%) | 98.90 | 21718 | 20244 |
|  | R2 | 16622150 | 16575017 | 15634734 (94.3%) | 98.86 | 21456 |  |
|  | R3 | 14666027 | 14636748 | 13879643 (94.8%) | 98.90 | 21249 |  |
